# Comparative Analysis of Six IRF Family Members in Alveolar Epithelial Cell-Intrinsic Antiviral Responses

**DOI:** 10.3390/cells10102600

**Published:** 2021-09-30

**Authors:** Sandra Wüst, Paulina Schad, Sandy Burkart, Marco Binder

**Affiliations:** 1 Research Group “Dynamics of Viral Infection and the Innate Antiviral Response”, Division F170, German Cancer Research Center (DKFZ), 69120 Heidelberg, Germany; s.wuest@dkfz.de (S.W.); schad@stud.uni-heidelberg.de (P.S.); s.burkart@dkfz.de (S.B.); 2 Faculty of Biosciences, Heidelberg University, 69120 Heidelberg, Germany

**Keywords:** innate immunity, antiviral response, interferon, cytokines, RIG-I-like receptors, RLR signaling, pattern recognition receptors, PRR signaling, transcription factors, IRFs

## Abstract

Host cell-intrinsic antiviral responses are largely mediated by pattern-recognition receptor (PRR) signaling and the interferon (IFN) system. The IFN regulatory factor (IRF) family of transcription factors takes up a central role in transcriptional regulation of antiviral innate immunity. IRF3 and IRF7 are known to be key players downstream of PRRs mediating the induction of type I and III IFNs. IFN signaling then requires IRF9 for the expression of the full array of interferon stimulated genes (ISGs) ultimately defining the antiviral state of the cell. Other members of the IRF family clearly play a role in mediating or modulating IFN responses, such as IRF1, IRF2 or IRF5, however their relative contribution to mounting a functional antiviral response is much less understood. In this study, we systematically and comparatively assessed the impact of six members of the IRF family on antiviral signaling in alveolar epithelial cells. We generated functional knockouts of IRF1, -2, -3, -5, -7, and -9 in A549 cells, and measured their impact on the expression of IFNs and further cytokines, ISGs and other IRFs, as well as on viral replication. Our results confirmed the vital importance of IRF3 and IRF9 in establishing an antiviral state, whereas IRF1, 5 and 7 were largely dispensable. The previously described inhibitory activity of IRF2 could not be observed in our experimental system.

## 1. Introduction

Invading pathogens, in particular viruses, are detected and fended off by a specialized and evolutionarily ancient part of the innate immune system. Germline encoded receptors for pathogen-associated molecular patterns (PAMPs), so called pattern recognition receptors (PRRs), are present in nearly all cells of the body. Upon sensing of viral components, PRRs mount conserved signaling cascades, eventually triggering the production and secretion of interferons (IFNs) and other cytokines that convey a danger signal to the infected cells and–importantly–to uninfected cells in the surrounding tissue. This signal then leads to the activation of a very broad transcriptional program involving the production of hundreds of IFN stimulated genes (ISGs) that tweak cellular homeostasis towards a condition still supporting cell survival but hostile to virtually any virus; this condition is termed the antiviral state of the host cell. As this process per se does not require the help of any professional immune cell, it is called cell-autonomous or cell-intrinsic innate immunity. Nonetheless, IFNs and cytokines secreted in the process do strongly act on immune cells, both innate and adaptive, playing the central role in orchestrating and coordinating all branches of the immune system. Especially upon infection with agents not encountered before in the life-time of an individual, pre-formed humoral or cellular immunity does not exist and cell-intrinsic responses become particularly decisive in determining the outcome of infection, as is observed in the currently raging SARS-CoV2 pandemic [1,2,3]. Our study is therefore explicitly limited to non-immune (epithelial) cells, investigating purely cell-intrinsic phenomena.

A multitude of different PRRs have been described to sense viral infection, ranging from Toll-like receptors in the plasma- or endosomal membrane to soluble cytosolic receptors such as cGAS or RIG-I-like receptors (RLRs). Despite this variety of PRRs, downstream signaling cascades are largely converging at the activation of a specific set of IκB-kinases, in turn leading to the activation of NF-κB and IRF transcription factors (for an overview, see Figure 8). While NF-κB is a major transcription factor for all antimicrobial PRR signaling cascades, additional triggering of IRF activity is defining the antiviral character of the response. In non-immune cells, the most important member of the IRF family downstream of PRRs is IRF3, which is expressed in virtually all cells of the body. Highly similar to IRF3 is IRF7, whose activation parallels that of IRF3, however, IRF7 is capable of inducing a broader range of type I IFNs, including the IFN-α subtypes, whereas IRF3 is largely restricted to inducing IFN-β and IFN-λ. IRF7 is rather lowly expressed in most cell types, but highly enriched in plasmacytoid dendritic cells, which, hence, are extraordinary producers of IFN-α upon PRR activation. Downstream of TLR7, 8, and 9, IRF5 (and not IRF3 or IRF7) has been shown to be crucially involved in the induction of IFNs [4,5], interestingly with a different profile in the induced gene expression program in human and murine systems [6,7,8]. IRF5 has also been implicated in IFN production downstream of RLR signaling [9], although this still is somewhat controversial. The produced IFN then signals through its cognate receptor (IFNAR1/2 for type I IFNs, IFNLR/IL10R2 for type III IFNs), eventually leading to the formation of the transcriptionally active hetero-trimeric factor ISGF3 [10]. ISGF3 is composed of (phosphorylated) STAT1 and STAT2, as well as IRF9, the latter of which is mediating the specificity of the transcription factor complex for ISRE-containing promoters of ISGs [11]. Another nuclear IRF, IRF2, has been described to antagonize the transcriptional activity of ISGF3 and dampen ISG production [12]. Type I IFN signaling also leads to other STAT containing transcription factor complexes, most notably STAT1 homodimers (also termed gamma-activated factor, GAF) [13] that are typically formed downstream of IFN-γ receptor signaling [14]. STAT1 homodimers lead to the expression of IRF1, which acts as a transcription factor trans-activating a plentitude of inflammatory and antiviral target genes [15]. It has been found to be important for the antiviral activity of IFN-γ and to a certain extent also type I IFN [16]. In type I IFN signaling, IRF1 also appears to promote proinflammatory responses, whereas type III IFN signaling does not lead to the induction of IRF1 [17]. In contrast to IRF3 and IRF7, IRF1 generally does not require post-translational activation in order to become transcriptionally active [15]. Like ISGF3 (IRF9), also IRF1-mediated transcriptional activation has been described to be inhibited by IRF2 [18]. Interestingly, the expression of IRF2 itself is induced both by type I/III IFN signaling (ISGF3, IRF9) as well as by IFN-γ (IRF1), suggesting it may play an important role as a negative feedback regulator (Figure 8). 

While for each individual IRF there are ample data available on its functional role in antiviral signaling and its molecular mechanism [10], there are few if any comprehensive studies addressing the functional importance of these transcription factors relative to each other. Our present study explicitly focused on epithelial cells, as they are primary targets of many viruses and their intrinsic antiviral system fulfills the role of a first line of defense against invading pathogens, well before professional immune cells are attracted and activated. We chose the human lung epithelial A549 adenoma cell line, widely used as a model for viral infection and to study antiviral responses. We have previously generated functional knockouts (KOs) of key genes of the antiviral system using CRISPR/Cas9 technology in these cells [19,20] and have now expanded our KO panel by IRF1, IRF2, IRF3, IRF5, IRF7, and IRF9. We studied the impact of these IRFs in PRR-triggered IFN production, focusing on the prototypic receptor RIG-I, as well as on type I and type III IFN signaling. Besides measuring the production of IFN or specific hallmark ISGs, we have also quantified the induction of a broad panel of cytokines and chemokines, as some IRFs, such as IRF1 and IRF5, have been implicated in proinflammatory programs. Lastly, we assessed the overall impact of IRF KO on the replication of two RNA viruses (Rift Valley fever virus and Influenza A virus) and two DNA viruses (Herpes Simplex virus 1 and Adenovirus type 5). Across all read-outs, we observed a massive impact upon KO of IRF3 or IRF9 as expected, as these factors assume central and essential roles in IFN induction and signaling, respectively. The effects of a functional lack of the other IRF family members were substantially milder or, in many cases, even absent. This was particularly surprising for IRF1, which was reported as a key antiviral factor, and IRF2, which was shown to significantly inhibit ISG induction and IRF1 activity. 

Our study is largely limited to RLR and IFN signaling in one alveolar epithelial cell line. Hence, it is well conceivable that in other cell types, especially in myeloid and other immune cell lineages, and for other PRR pathways, such as TLRs or cytosolic DNA sensors, IRFs dependencies may differ considerably. Nonetheless, our study provides– to our knowledge– the most comprehensive and systematic comparative analysis of IRF function in cell-intrinsic antiviral signaling of epithelial cells.

## 2. Materials and Methods

### 2.1. Cell Lines

All cell lines were cultured in Dulbecco’s modified Eagle medium (DMEM, 41965062, Thermo Fisher Scientific) containing 10% fetal bovine serum (10270106, FBS, Sigma Aldrich), 100 µg/mL penicillin, 100 µg/mL streptomycin (15140122, Thermo Fisher Scientific) and 1% non-essential amino acids (11140050, Thermo Fisher Scientific). All infections were performed in DMEM containing 2% fetal bovine serum, 100 µg/mL penicillin, 100 µg/mL streptomycin and 1% non-essential amino acids, further referred to as DMEM_INF_. Cell lines were cultured at 37 °C in an incubator at 95% humidity and 5% CO_2_. A549, HEK293T, Vero E6, and BHK-21 cells were obtained from Ralf Bartenschlager at the University Hospital Heidelberg, Germany. MDCKII cells were generously provided by Martin Schwemmle, University Hospital Freiburg, Germany. A549 IRF1, IRF2, IRF3, IRF5, IRF7, IRF9, RIG-I, RELA, and IFNAR1/IFNLR/IFNGR1 (further referred to as IFNRTKO) knockout cell lines were generated by us using the CRISPR/Cas9 technology and are derived from a single cell clone. The IRF3 KO [21], RIG-I KO [19,20,22] and MAVS KO [20] clones were published previously.

#### 2.1.1. Generation of A549 CRISPR/Cas9 Knockout Cell Lines

CRISPR guide RNA sequences targeting IRF1, IRF2, IRF3, IRF5, IRF7, IRF9, RIG-I, RELA, IFNAR1, IFNLR or IRFNGR1 (see Table 1) were designed using E-CRISP [23], ordered as dsDNA oligonucleotides and introduced into the BsmBI site of the lentiCRISPR v2 plasmid (#52961, Addgene). As a negative control, a human genome non-targeting guide RNA sequence (further referred to as NT) was chosen (sequence taken from the GeCKO CRISPR v2 library [24]). Final lentiCRISPR v2 plasmids with inserted guide RNA sequences were validated by Sanger sequencing. CRISPR/Cas9 knockout pools were generated by lentiviral transduction. The production of lentiviral particles was accomplished by calcium phosphate transfection of HEK293T cells with three plasmids used at a 3:1:3 (per mass) ratio: (1) pCMV-ΔR8.91, encoding HIV gag-pol; (2) pMD2.G, coding for VSV-G glycoprotein; and (3) the respective lentiCRISPR v2 plasmid. Supernatants containing lentiviral particles were harvested 48, 56, and 72 h post-transfection, and used to transduce A549 cells at the given time points. Transduced cells were selected by supplementing 1 µg/mL puromycin (P7255-100MG, Sigma Aldrich) to the cell culture medium. Selected pools were validated (see 2.1.2 Validation of of CRISPR/Cas9 Knockouts) and single-cell clones were seeded by limiting dilution of 1 cell per 2 wells in 96-well plates. Once a sufficient cell density was reached, the single-cell clones were again validated for the successful knockout of the desired genes.

#### 2.1.2. Validation of CRISPR/Cas9 Knockouts

##### Validation by Immunoblotting

Untreated or IFN-α2a stimulated (200 IU/mL; 11100-1, PBL Assay Science) A549 IRF1, IRF2, IRF3, IRF5, IRF7, IRF9, RIG-I, and RELA KO cells were washed with PBS and directly lysed in Laemmli sample buffer (0.1 M TRIS [pH 6.8], 30% glycerol, 3% SDS, 7.5% b-mercaptoethanol, 0.06% bromophenol blue) (see 2.6.2 Read-out by Immunoblotting). See also Supplementary Figure S1A,B.

##### Validation of Functional Knockout (Quantitative RT-PCR)

To validate the loss of function in A549 IRF3, IRF9 and RELA KO cell lines the transcripts of IFIT1, IFNB1 (IFN-β), and CCL5 were measured after treatment with IFN-α2a (200 IU/mL) or transfection with 5’ppp-dsRNA (2 ng/well; 200 bp length, homemade). For validation of the A549 IRF7 KO clone, cells were pre-treated overnight with IFN-β to induce IRF7 expression, and then stimulated by transfection with 5’ppp-dsRNA. IFN-α transcripts were measured by qRT-PCR. For validation of the A549 IFNAR1/IFNLR/IFNGR1 (IFNRTKO) the cells were stimulated with INF-α2a (200 IU/mL), IFN-λ1 (5 ng/mL; 300-02L-100, Peprotech), and IFN-γ (200 IU/mL; 285-IF-100/CF, R&D Systems) and IFIT1 transcripts were measured (see 2.6.3 Read-out by qRT-PCR). See also Supplementary Figure S1C.

##### Validation by Sequencing of Genomic Loci

Genomic DNA was prepared from single-cell KO clones of IRF1, IRF2, IRF3, IRF5, IRF7, IRF9, RELA, and RIG-I. PCR was performed using primer pairs surrounding the sgRNA binding site, and amplicons were purified and subjected to next-generation sequencing. Indels were analyzed with IGV [25]. See also Supplementary Figure S1D.

#### 2.1.3. Generation of A549 Overexpression Cell Lines

The plasmids pENTR221_IRF1 and pENTR221_IRF5 were taken from the ORFeome Collaboration [26]. IRF7 was amplified with gene specific primers flanked by attB sites (forward sequence: GGGGACAAGTTTGTACAAAAAAGCAGGCTTCatggccttggctcctgagagg; reverse sequence: GGGGACCACTTTGTACAAGAAAGCTGGGTCctaggcgggctg¬ctccagctc) from a plasmid kindly provided by Friedemann Weber (University of Gießen, Germany), and shuttled into the entry vector pDONR207 using the Gateway Cloning Technology [27]. After validation by Sanger sequencing, all three genes were shuttled into the destination vector pWPI_nHA_Blr by Gateway Cloning. The production of lentiviral particles was accomplished by calcium phosphate transfection as described above (see 2.1.1 Generation of A549 CRISPR/Cas9 Knockout Cell Lines). As control, the cells were transduced with empty pWPI_nHA-Blr vector. To select for successfully transduced cells, 5 µg/mL blasticidin S hydrochloride (15047750, MP Biomedicals) was added to the medium.

##### Validation of IRF1, IRF5, and IRF7 Overexpression by Immunoblotting

1 × 10^6^ A549 empty-nHA, IRF1-nHA, IRF5-nHA, and IRF7-nHA overexpression cells were harvested and further analyzed by staining for the HA-tag (see 2.6.2 Read-out by Immunoblotting). See also Supplementary Figure S7.

##### Validation of IRF1, IRF5, and IRF7 Overexpression by Quantitative RT-PCR

4 × 10^5^ A549 empty-nHA, IRF1-nHA, IRF5-nHA, and IRF7-nHA overexpression cells were harvested and IRF1, IRF5, and IRF7 transcripts were measured (see 2.6.3 Read-out by qRT-PCR). See also Supplementary Figure S7.

### 2.2. Virus Stock Production and Titration

#### 2.2.1. Influenza A SC35M Virus Stock Production

MDCK-II Madin-Darby canine kidney strain II cells were seeded on 15 cm cell culture dishes reaching approximately 80% confluency at the time point of infection. Cells were infected with Influenza A SC35M Gaussia luciferase reporter virus (SC35MNS1_2A_GLuc_2A_NEP [28], generously provided by Martin Schwemmle, University Hospital Freiburg, Germany) at an MOI of 0.01 in DMEM_INF_. As soon as a complete cytopathic effect was observable (2–3 days post infection), supernatants were transferred into falcon tubes and centrifuged at 1000 rpm for 10 min at 4 °C to clear the viral supernatants from cell debris. The supernatants were aliquoted, stored frozen at −80 °C and subsequently titrated by plaque assay and TCID_50_ (see 2.6.1 Read-out by Luciferase Assay) on MDCK-II cells. 

#### 2.2.2. Rift Valley Fever Virus Stock Production

Vero E6 African green monkey kidney epithelial cells were seeded on 15 cm cell culture dishes reaching approximately 80% confluency at the time of infection. The cells were infected with Rift Valley fever virus harboring a Renilla luciferase in place of NSs (RVFVΔNSs_RLuc [29], kind gift of Friedemann Weber, University of Gießen, Germany) at an MOI of 0.001 in DMEM_INF_. As soon as a complete cytopathic effect was observable (2–3 days post infection), supernatants were transferred into falcon tubes and centrifuged at 1000 rpm for 10 min at 4 °C to clear the viral supernatants from cell debris. The supernatants were aliquoted, stored frozen at −80 °C and subsequently titrated by plaque assay and TCID_50_ (see 2.6.1 Read-out by Luciferase Assay) on Vero E6 cells.

#### 2.2.3. Adenovirus Stock Production

A549 cells were seeded on 15 cm cell culture dishes reaching approximately 80% confluency at the time of infection. The cells were infected with Adenovirus stock (AdΔfiberIL [30], kindly provided by Dirk Nettelbeck, German Cancer Research Center (DKFZ), Germany) of passage 3. As soon as a partial cytopathic effect was observable (48 h post-infection), cells were scraped off the dish and together with the supernatant were transferred into falcon tubes and centrifuged at 2000 rpm for 30 min at 4 °C. The cell pellets were re-suspended in lysis buffer (10 mM TRIS [pH 8], 135 mM NaCl, 1 M MgCl_2_] and purified by cesium-chloride density gradient ultracentrifugation at 38,000 rpm for 3 h at 10 °C. The purified virus was collected and mixed in a 1:1 ratio with glycerol storage solution (10 mM TRIS [pH 8], 100 mM NaCl, 1 M MgCl_2_, 0.1% BSA, 50% Glycerol). The virus solution was aliquoted, frozen, and stored at −80 °C. Titration of the virus stock was performed by OD_260_ reading and TCID_50_ (see 2.6.1 Read-out by Luciferase Assay) on A549 cells.

#### 2.2.4. Herpes Simplex Virus 1 Stock Production

BHK-21 baby hamster kidney cells were seeded on 15 cm cell culture dishes reaching approximately 90% confluency at the timepoint of infection. The cells were infected with Herpes simplex virus 1 encoding mCherry and *Gaussia* luciferase (HSV1(17^+^)Lox-CheP2AGLuc [31], generously provided by Beate Sodeik, Hannover Medical School, Germany) at an MOI of 0.01 in DMEM_INF_. As soon as a complete cytopathic effect was observable (2–3 days post-infection), supernatants were transferred into falcon tubes and centrifuged at 4000 rpm for 10 min at 4 °C to clear the viral supernatants from cell debris. Afterwards, a second centrifugation step at 12,000 rpm for 90 min at 4 °C was performed. The supernatants were aliquoted, frozen, stored at −80 °C, and subsequently titrated by plaque assay and TCID_50_ (see 2.6.1 Read-out by Luciferase Assay) on A549 cells.

#### 2.2.5. Virus Stock Titration by Plaque Assay

Cells were seeded into 12-well cell culture plates reaching approximately 80% confluency at the time of infection. Ten-fold dilution series of the virus stock, starting with 1:100, were applied onto the cells and incubated for 1 h at room temperature while gently shaking. After 1 h the inoculum was replaced by fresh DMEM_INF_ containing 1.5% cellulose powder (Avicel^®^ RC-591 NF, Dupont). After 3 days, the medium was removed and the cells were gently washed twice with 1x PBS. 1 mL per well of 4% paraformaldehyde was applied for 10 min to fix the cells. After removal of the fixation solution, staining was performed with 1% crystal violet in 10% ethanol followed by several washing steps with destilled-H_2_O. Plaques were counted and the titer was determined by considering the dilution steps to give plaque forming unit per mL (PFU/mL).

#### 2.2.6. Virus Stock Titration by TCID_50_ Luciferase Assay

Cells were seeded in a 96-well plate at a density of 7 × 10^3^ cells per well. In a second 96-well plate 1:2 serial dilutions of the virus stocks, starting with a pre-dilution of 10^−2^, were set up in DMEM_INF_. Cells were washed once with PBS and the virus dilutions were transferred onto the cells. 72 h post infection cells were washed once with PBS and lysed with 25 μL per well of luciferase lysis buffer (1% Triton X-100, 25 mM glycyl-glycin [pH 7.8], 15 mM MgSO_4_, 4 mM EGTA, 10% glycerol [99%]) containing 1 mM DTT and stored frozen at −80 °C until further analysis by luciferase assay (see 2.6.1 Read-out by Luciferase Assay)

### 2.3. Virus Infections

#### 2.3.1. Rift Valley Fever Virus Infection

A549 IRF1, 2, 3, 5, 7, 9 RIG-I KO clone, and A549 NT cells were seeded in a 24-well plate at a density of 0.5 × 10^5^ cells per well and infected the next morning with Rift Valley fever reporter virus (RVFVΔNSs_RLuc) at an MOI of 0.01 in DMEM_INF_. Supernatants were harvested 0, 8, 24, and 32 h post infection. The cells were washed once with PBS and were directly lysed in 100 μL per well with luciferase lysis buffer containing 1 mM DTT at the given time points. The supernatants and cell lysates were stored frozen at −80 °C until further analysis (see 2.6.1 Read-out by Luciferase Assay).

#### 2.3.2. Influenza A SC35M Virus Infection

A549 IRF1, 2, 3, 5, 7, 9 RIG-I KO clone, and A549 NT cells were seeded in a 24-well plate at a density of 0.5 × 10^5^ cells per well and infected the next morning with Influenza A SC35M reporter virus (SC35MNS1_2A_GLuc_2A_NEP) at an MOI of 0.001 in DMEM_INF_. Supernatants were harvested 0, 8, 24, 32, 48, and 56 h post infection. The supernatants were stored frozen at −80 °C until further analysis (see 2.6.1 Read-out by Luciferase Assay).

#### 2.3.3. Adenovirus Infection

A549 IRF1, 2, 3, 5, 7, 9 RIG-I KO clone, and A549 NT cells were seeded in a 24-well plate at a density of 0.5 × 10^5^ cells per well (for 0–56 h post infection) or at a density of 0.25 × 10^5^ cells per well (72–96 h post infection) and infected the next morning with Adenovirus (AdΔfiberIL_FLuc) at an MOI of 0.05 in DMEM_INF_. Supernatants were harvested 0, 8, 24, 32, 48, 72, and 96 h post infection. The cells were washed once with PBS and were directly lysed in 100 μL per well of luciferase lysis buffer containing 1 mM DTT at the given time points. The supernatants and lysed cells were stored frozen at −80 °C until further analysis (see 2.6.1 Read-out by Luciferase Assay).

#### 2.3.4. Herpes Simplex Virus 1 Infection

A549 IRF1, 2, 3, 5, 7, 9 RIG-I KO clone, and A549 NT cells were seeded in a 24-well plate at a density of 0.5 × 10^5^ cells per well and infected the next morning with Herpes simplex virus 1 (HSV1(17^+^)Lox-CheP2AGLuc) at an MOI of 0.1 in DMEM_INF_. Supernatants were harvested 0, 8, 24, 32, 48, and 56 h post infection. The supernatants were stored frozen at −80 °C until further analysis (see 2.6.1 Read-out by Luciferase Assay).

### 2.4. IFN Stimulation

A549 IRF1, 2, 3, 5, 7, 9, RIG-I KO clone, and A549 NT cells were seeded in a 24-well plate at a density of 1 × 10^5^ cells per well and stimulated with 500 IU/mL IFN-β (8499-IF-010/CF, R&D Systems) or 5 ng/mL IFN-λ1. The cells were washed once with PBS, directly lysed with 350 μL Monarch™ RNA Lysis Buffer (T2012L, NEB) per well at 8 and 24 h post stimulation and stored frozen at −80 °C until further analysis (see 2.6.3 Read-out by Quantitative RT-PCR).

### 2.5. dsRNA Stimulation

A549 IRF1, 2, 3, 5, 7, 9, RIG-I KO clone, and A549 NT cells were seeded in a 24-well plate at a density of 1 × 10^5^ cells per well and transfected with 2 ng 5’ppp-dsRNA per well using Lipofectamine2000 (11668019, Thermo Fisher Scientific) according to the manufacturer’s protocol. As control, non-stimulating poly(C) (P4903-10MG, Sigma Aldrich) was used at a concentration of 500 ng per well. To be consistent in the amount of transfected 5’ppp-dsRNA per 1 μL Lipofectamine 2000, 1 ng 5’ppp-dsRNA was mixed with 499 ng poly(C) to reach 500 ng per well. Supernatants were harvested 4, 8, 24, 48, and 72 h post stimulation. The cells were washed once with PBS, directly lysed with 350 μL Monarch™ RNA Lysis Buffer per well at the given time points and stored frozen at −80 °C until further analysis.

### 2.6. Read-Out Methods

#### 2.6.1. Read-Out by Luciferase Assay

Previously described reporter viruses were used to directly measure replication over time. *Firefly* luciferase signal was measured by injecting 400 μL per well of luciferase assay buffer (15 mM K_3_PO_4_ [pH7.8], 25 mM glycyl-glycin [pH 7.8], 15 mM MgSO_4_, 4 mM EGTA) supplemented with 1 mM DTT, 2 mM ATP, and 1 mM D-Luciferin (102111, PJK, Germany) followed by read-out of the signal without filter. *Renilla* and *Gaussia* luciferase signal was measured by injecting 400 μL luciferase assay buffer supplemented with 1.43 mM coelenterazine (102173, PJK, Germany) into each well followed by read-out of the signal with a 480 nm high-sense filter (480m20BREThs, Berthold). After the measurement of each well the luciferase signal was immediately extinguished by injection of 100 μL 10% SDS per well before the next measurement. Luciferase reporter assays were measured with the Mitras2 multimode plate reader (LB942, Berthold).

#### 2.6.2. Read-Out by Immunoblot

Cells were directly lysed in Laemmli sample buffer (0.1 M TRIS [pH 6.8], 30% glycerol, 3% SDS, 7.5% b-mercaptoethanol, 0.06% bromophenol blue) and boiled 5 min at 95 °C for denaturation. Proteins were separated on 10% SDS-PAGEs, transferred onto PVDF membranes (1620177, Bio-Rad) and stained for IRF1, IRF2, IRF3, IRF5, IRF7, IRF9, RIG-I, RELA, and Calnexin (see Table 2). Secondary HRP conjugated goat anti-mouse (1:10,000 dilution) or anti-rabbit (1:20,000 dilution) antibodies (Sigma Aldrich) were used to detect the primary antibodies. Clarity Western ECL Blotting Substrates (170-5061, Bio-Rad) or ECL Prime Western Blotting System (RPN2236, GE Healthcare Life sciences) were used to incubate the membranes and chemo luminescence was detected by a high-sensitivity CCD camera (ChemoCam Imager 3.2, INTAS, Germany). Images were taken without binning at 16 bit/pixel. LabImage 1D software (INTAS/KAPELAN, Germany) was used for the quantification of luminescent signals of immunoblots. Signals of the target proteins were normalized to the respective loading controls.

#### 2.6.3. Read-Out by Quantitative RT-PCR

Transcript levels of IRF1, IRF2, IRF3, IRF5, IRF7, IRF9, IFNAR1, IFIT1, MX1, IFN-β, RANTES (CCL5), and TNFAIP3 (A20) genes were measured by quantitative RT-PCR. According to the manufacturer’s protocol total RNA was isolated from cell lysates using the Monarch Total RNA Miniprep Kit (T2010S, NEB). Reverse transcription was performed using the High-Capacity cDNA Reverse Transcription Kit (43-688-14, Applied Biosystems) following the manufacturer’s protocol in a thermal cycler (C1000 Touch™, Bio-Rad). Gene specific primers (see Table 3) with exon-exon junction overlap designed with PrimerBlast [32] and iTaq Universal SYBR Green Supermix (17251525, Bio-Rad, Germany) were used to perform quantitative RT-PCR. Relative transcript levels were determined using only the ΔC_T_ (relative to GAPDH), and not normalizing to mock / unstimulated controls as in the ∆∆C_T_ method. This was favorable as the expression of many genes of interest (e.g., IFNB1) is minute and not reliably measurable in the absence of stimulation, and normalization to such unreliable background values close to zero would introduce a high degree of artificial noise.

#### 2.6.4. Read-Out by MSD Electroluminescent Multiplex Assay

In order to assess released IFN, chemokine, and proinflammatory cytokine profiles, three commercially available kits for the Mesoscale multiplex platform were used. Specifically, the U-PLEX Interferon Combo (hu) (K15094K-1) was used to detect IFN-α2a, IFN-β, IFN-γ, and IL-29/IFN-λ1 in supernatants of uninfected and infected cells with the previously described viruses. For the measurement of chemokine and proinflammatory cytokine levels in supernatants of unstimulated and 5’ppp-dsRNA-stimulated cells, V-PLEX Chemokine Panel 1 Human Kit (K15047D-1) was used for the determination of Eotaxin, Eotaxin-3, IL-8, IL-8 (HA), IP-10, MCP-1, MCP-4, MDC, MIP-1α, MIP-1β, and TARC, whereas the V-PLEX Proinflammatory Panel 1 Human Kit (K15049D-1) covered the analytes IFN-γ, IL-1β, IL-2, IL-4, IL-6, IL-8, IL-10, IL-12p70, IL-13, and TNF-α. The assays were read out by the MSD Quickplex SQ120 device and the results evaluated with the Methodical Mind and MSD Discovery Workbench Version 4.0 software. All kits were used according to manufacturer’s instructions. Note that based on the standards employed in each assays, the software determined upper and lower limits of quantification (ULoQ and LLoQ), beyond which measurements are still possible but cannot be reliably quantified; we included these values in the graphs but clearly indicate the U/LLoQ in the figure. Individual samples below the limit of detection (LoD) are identified by missing symbols in the graphs, or denoted as “not detectable” (n.d.) if all replicates were below the LoD.

### 2.7. Statistics

If not denoted otherwise, experiments have been performed three times independently. Wherever possible and meaningful, we show all independent replicates as individual data points (technical replicates, e.g., multiple wells, from one experiment were averaged), with same symbols representing the same experiment; this provides direct and accessible information about the reproducbility of trends and effects. We have omitted results of statistical testing in the text and figures, as significance thresholds (e.g., α = 0.05) tend to convey a yes/no message that may not be warranted when judging minor effects. Results of a multiple ratio paired *t*-test analysis with correction for multiple testing by the Holm-Šídák method for Figures 5–8 are reported in Supplementary File “statistics.xlsx”); note that not detected values (measurements below the detection limit) preclude proper (pair-wise) testing. Testing was performed using the GraphPad Prism (v9) software.

## 3. Results

### 3.1. Generation of Knockout Cell Lines and Regulation of IRF Expression

**Figure 1:**
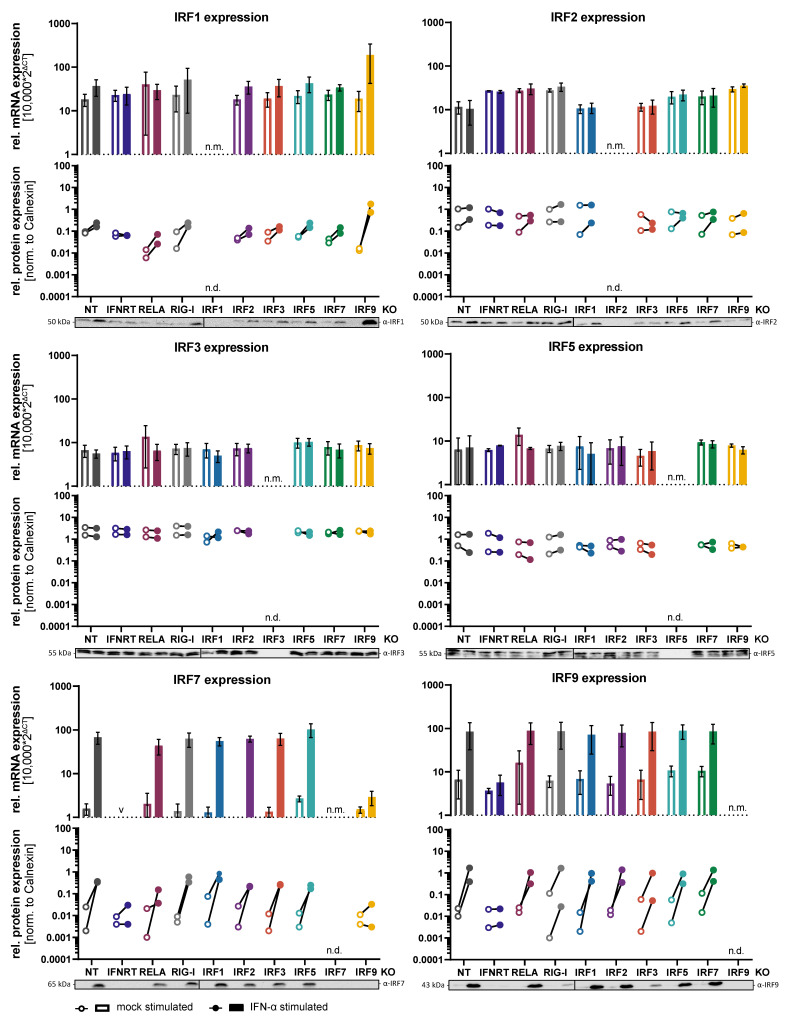
Baseline and IFN stimulated expression of IRFs and their mutual dependence on other IRFs. A549^NT^ (non-targeting CRISPR control), A549^IFNR TKO^ (IFNAR1, IFNGR1, IFNLR triple KO), A549^RELA KO^ (RELA/NF-κB p65 KO), A549^RIG-I KO^ (RIG-I KO), and A549^IRF (as indicated) KO^ were mock treated (empty bars and symbols) or treated with IFN-α (200 IU/mL) for 16 h (solid bars and symbols). The expression of IRF1, -2, -3, -5, -7, and -9 was determined at mRNA level by qRT-PCR (relative to GAPDH, top half of panels), mean ± SD of three independent experiments is shown. IRF expression at the protein level was determined by immunoblotting (lower half of panels). For protein quantification, immunoblot ECL signals were measured by a CCD instrument and normalized to the respective signal of the loading control (calnexin). Quantifications of two independent experiments are shown and one of the two experiments is shown as an image at the bottom; see also Supplementary Figure S1. Protein and mRNA levels of the respective knocked-out IRFs were not determined (“n.d.”).

A549 cells are human adenocarcinoma cells of lung (alveolar) epithelial origin, which feature sensitive virus sensing pathways and potent antiviral signaling. We have previously successfully established knockouts (KOs) of various components of the cell-intrinsic antiviral signaling system in A549 cells, such as RIG-I [19,20,22], MAVS [20], and IRF3 [21], and could demonstrate that such KOs completely abrogate IFN production upon RIG-I stimulation by virus infection or dsRNA transfection. In the present study, we now addressed the question if and to what extent other IRFs contribute to the mounting of a full-fledged antiviral response in epithelial cells. We focused on those genes whose transcriptional expression could be detected in A549 cells: IRF1, IRF2, IRF3, IRF5, IRF7, and IRF9; these are also the genes previously implicated with cell-intrinsic immune responses in non-professional immune cells. We generated functional KOs of these IRFs by CRISPR/Cas9 technology and selected single cell clones that showed no remaining expression of the respective protein in immunoblot analysis (Figure 1, Supplementary Figure S1) and, where feasible, could be confirmed by functional assays and next generation sequencing of the respective genomic locus (Supplementary Figure S1C,D). As controls, we included non-targeting CRISPR gRNA (“NT”), KO of RIG-I, a triple-KO of all IFN receptors (IFNAR1/IFNGR1/IFNLR; “IFNRT”), and KO of the NF-kB subunit RELA. We then investigated the expression of the IRFs upon type I IFN treatment both on mRNA and protein level (Figure 1). We found IRF7 to be most strongly upregulated by type I IFN, but also IRF9 was robustly induced. IRF1 was only minimally induced, which is consistent with the notion of it being a type II rather than type I IFN responsive gene. As expected, IFN treatment in cells lacking the IFN receptors (IFNRT) resulted in no IRF induction, whereas lack of RELA or RIG-I had no effect. Albeit expressed and detectable at baseline, we observed no further induction upon IFN treatment for IRF2, -3 and -5, with a slight tendency towards increased protein levels in case of IRF2 (Figure 1). We have furthermore assessed expression of the IRFs in each KO cell line to investigate potential mutual regulation. In general, there was no notable effect of any KO onto baseline levels of any other IRF, neither at mRNA nor at protein level. Additionally, expression upon IFN-α treatment was mostly unaffected by the IRF KOs, with the exception of IRF9 KO. As expected, IRF9 KO almost completely abolished the induction of IRF7, which is a well-known ISG induced by the heterotrimeric transcription factor complex ISGF3, of which IRF9 is a crucial component [10]. Interestingly, however, in case of IRF1 expression, the KO of IRF9 even markedly augmented the induction upon IFN-α treatment, both at mRNA as well as protein level (Figure 1). Likely, this effect was also due to the inability of ISGF3 to form in response to IFN-α stimulation, leading to preferential formation of STAT1 homodimers, the major IFN-γ activated transcription factor inducing the expression of IRF1.

In addition to type I IFN treatment, we have also assessed the induction of IRF expression in response to RIG-I stimulation by infection with an attenuated variant (∆NSs) of Rift Valley fever virus (RVFV). This will lead to triggering of RLR/MAVS/IRF3 signaling, resulting in the production and secretion of type I and III IFN and auto- and paracrine IFN signaling (for reference see Figure 8). Overall, the induction pattern of IRFs was highly reminiscent of that upon IFN-α stimulation (Supplementary Figure S2). IRF9 was induced comparably, whereas IRF1 and IRF7 were induced even stronger than by the above described 200 IU/mL IFN-α treatment. Notably, upon virus infection, also IRF2 was induced, both at mRNA and protein level. This increased induction of IRF1, -2, and -7 may either indicate that RVFV infection led to the production of higher total levels of IFN, or that these genes can additionally be directly induced by IRF3 downstream of the RLR signaling cascade. Indeed, KO of IRF3 abolished the induction of IRF1, -2, 7, and -9. However, also KO of the IFN receptors (IFNRT) did so, rather arguing for a fully IFN dependent effect. In line with IFNRT, also KO of IRF9 again prevented the upregulation of IRF7 and also IRF2, but peculiarly, different from in the infection setting it did not lead to augmentation of IRF1 induction (Supplementary Figure S2).

### 3.2. Impact of IRFs on the Induction of Antiviral Response Gene Expression

**Figure 2:**
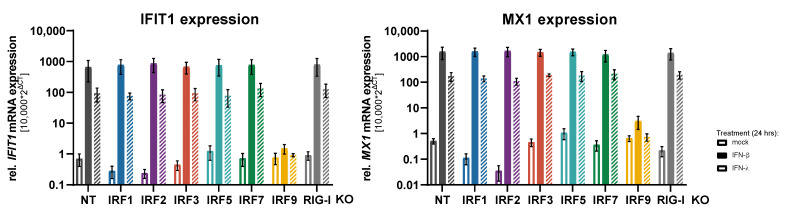
Impact of IRF KO on ISG induction upon IFN treatment. A549^NT^, A549^IRF (as indicated) KO^, and A549^RIG-I KO^ cells were stimulated with IFN-β (500 IU/mL) or IFN-λ (5 ng/mL) for 24 h. Relative IFIT1 and MX1 mRNA levels were measured by qRT-PCR. Data show the mean ± SD of three independent experiments.

As transcription factors, IRFs may be involved in the proper induction of the response genes downstream of pattern recognition receptors (PRRs) and of IFN receptors. First, we assessed the expression of two classical ISGs, IFIT1 and MX1, upon type I or type III IFN treatment across the different IRF KO cell lines. By and large, there was no substantial difference in baseline expression of these two genes amongst all IRF KOs. Notably, a lack of IRF2, which was described as a negative regulator of ISG expression, did not increase ISG levels, but–if anything–rather decreased them (Figure 2). Likewise, treatment with IFN-β or IFN-λ led to a massive induction of IFIT1 and MX1 mRNA levels, which was not affected by the lack of any of the tested IRFs, except for IRF9 once again abrogating ISGF3 formation and, hence, type I/III IFN signaling. Again, this was also true for IRF2, indicating that at this dosing (500 IU/mL IFN-β, 5 ng/mL IFN-λ) and timing (24 h treatment) of the IFN treatment, IRF2 does not negatively affect ISG expression in A549 cells. We therefore also tested a shorter IFN treatment of only 8 h, but could not observe any differences in ISG induction among the IRF KOs (apart from IRF9) (Supplementary Figure S3).

**Figure 3:**
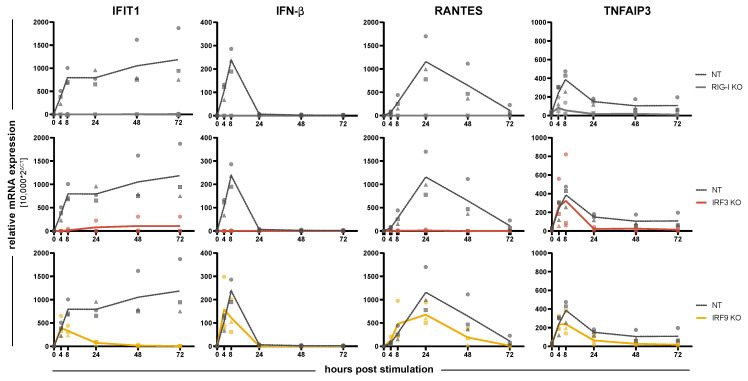
Impact of IRF KO on the induction kinetics of response gene expression. A549^NT^, A549^RIG-I KO^, A549^IRF3 KO^, and A549^IRF9 KO^ cells were stimulated by transfection of 5’ppp-dsRNA (1 ng/well) and relative IFIT1, IFN-β, RANTES (CCL5), and TNFAIP3 mRNA levels were measured at 4, 8, 24, 48, and 72 h post stimulation (h.p.i.) by qRT-PCR. Measurements (mean of technical triplicates) of three independent experiments are shown as symbols; means of the three experiments are connected by lines to visualize the time course.

**Figure 4:**
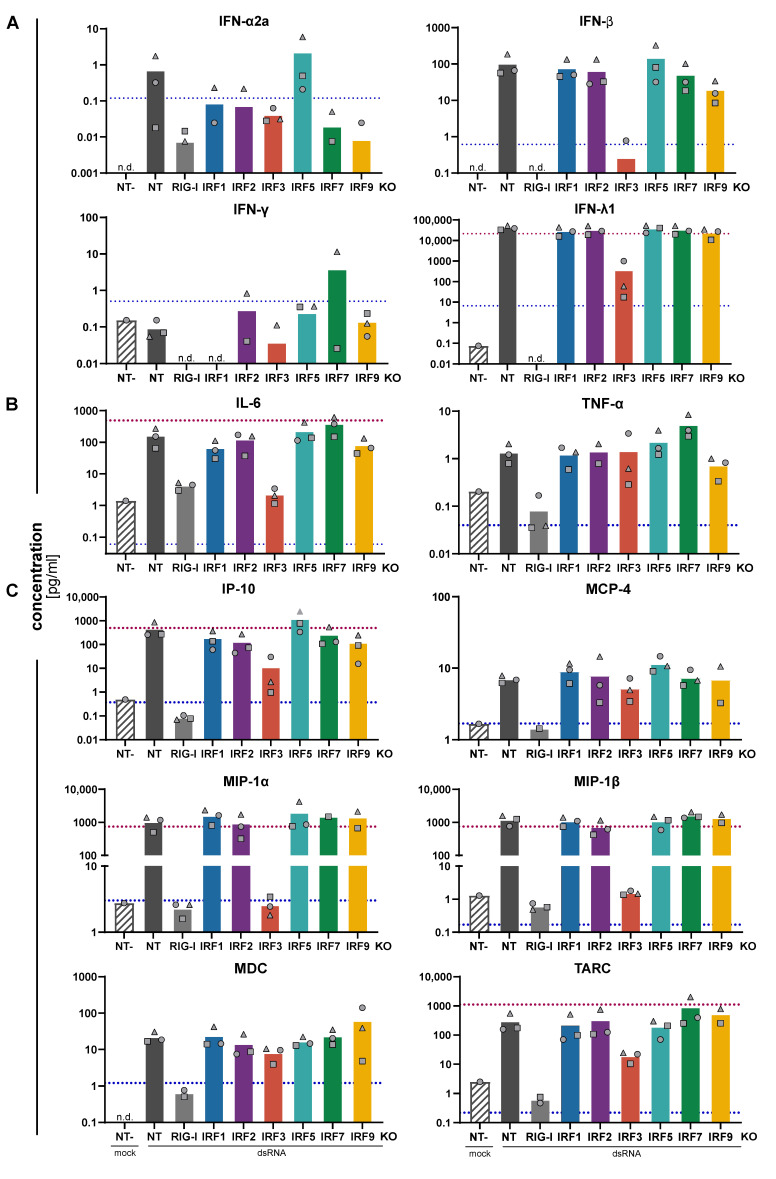
Cytokine and chemokine production of IRF KO cell lines upon stimulation of RIG-I. A549^NT^, A549^RIG-I KO^, and A549^IRF (as indicated) KO^ cells were stimulated by transfection of the RIG-I agonist 5’ppp-dsRNA (1 ng/well) for 24 h. Concentrations of indicated (**A**) IFNs, (**B**) proinflammatory cytokines and (**C**) chemokines in the cell culture supernatants were assessed by electroluminescent multiplex assays. “NT-” denotes the unstimulated (mock transfected) control condition. Bars represent the mean of three biologically independent experiments, with the three individual data points shown as gray symbols (same shapes represent same experiment); missing symbols indicate “non-detectable” measurements. Dashed lines represent the lower (blue) and upper (red) limit of quantification of the assay.

We next went on to investigate a possible impact of IRFs on the induction kinetics of response genes downstream of RLR signaling, including auto- or paracrine IFN signaling. In order to assess the kinetics of gene induction and to exclude effects of virus infection kinetics, we employed transfection of cells with a well-defined, purified RIG-I ligand (5’ppp-dsRNA) for RLR stimulation. As read-out, we measured mRNA levels over time (0, 4, 8, 24, 48, and 72 h) for four different genes: IFN-β (IFNB1), the primary response gene of the RLR system and unaffected by IFN signaling; RANTES (CCL5), another early response gene (cytokine) directly dependent on IRF3; TNFAIP3 (also known as A20), a direct response gene of NF-κB signaling downstream of RIG-I and independent of IRF3 and IFN signaling; and IFIT1, a well-known early ISG directly induced by IRF3 downstream of RLR signaling as well as by ISGF3 downstream of the IFN receptors. In control (NT) cells, we could observe the expected induction dynamics (Figure 3). IFN-β was induced very early (peak: 8 h) but very transiently upon 5’ppp-dsRNA transfection, returning to baseline by 24 h; RANTES reached its maximum expression only after 24 h, but returned to baseline by 72 h; TNFAIP3 was induced similarly to IFN-β, also peaking at 8 h already, but it sustained approximately half-maximal levels throughout the experiment; lastly IFIT1 was also quickly induced (8 h), but maintained high expression levels and even increased between 24 and 72 h, likely mediated by the secreted and accumulating IFNs (Figure 3); this is supported by high levels of IFN-β protein measured in the cells’ supernatants (Figure 4). As expected, due to the highly specific nature of the stimulus, none of the response genes was induced in RIG-I KO cells; only for TNFAIP3 a slight increase could be observed early after transfection, hinting towards a minute amount of NF-κB activation through RIG-I independent pathways. KO of IRF3, on the other hand, also abolished IFN-β (mRNA and protein, see Figure 3 and Figure 4) and RANTES induction and strongly diminished IFIT1 expression. TNFAIP3, however, was induced comparably to control cells, demonstrating that loss of IRF3 does not impact RLR signaling in general and leaves NF-κB activation untouched (Figure 3). KO of IRF9 was expected to affect IFN signaling, as it is a critical constituent of ISGF3, but should not impact on signaling upstream of IFN (i.e., the RLR pathway). Indeed, IRF9 KO did not significantly alter the induction kinetics of IFN-β, RANTES and TNFAIP3. For IFIT1, the early transcriptional induction– likely driven by IRF3 activity– was hardly affected, whereas the sustained and even increasing expression over the time course was abrogated, returning to negligible baseline levels from 24 h on and qualitatively resembling the expression kinetics of IFN-β (Figure 3).

As for KO of IRF1, -2, -5, and -7, we observed no clear and reproducible impact on the expression kinetics of the four tested response genes (Supplementary Figure S4). This included IRF2, which was reported to inhibit ISRE-mediated transcription, but while IFN-β expression tended to be pronounced in IRF2 KO (also in IRF1 KO), there was no such tendency for the ISG IFIT1. However, it must be noted that variability across the (biologically independent) experiments was prominent, making it difficult to pinpoint less pronounced effects.

### 3.3. Impact of IRFs on the Expression Pattern of Cytokines and Chemokines

Besides direct triggering of a transcriptional program that induces a functionally antiviral state of the cell, another physiologically important facet of the PRR-mediated antiviral response of tissue cells is the production and secretion of cytokines and chemokines. Apart from type I and III IFN, which primarily (but not exclusively) function by reinforcing the antiviral state of the infected cells and communicating this state to uninfected bystanders, further cytokines and, in particular, chemokines are produced. These extracellular messengers establish the communication with professional immune cells both of the innate as well as of the adaptive branch of the immune system. To this end, we stimulated our A549 IRF KO cells with RIG-I-specific 5’ppp-dsRNA and collected the supernatants 24 h post-stimulation. The supernatants were then analyzed for the presence of a broad panel of cytokines and chemokines by multiplexed assays employing electroluminescent read-out. As expected, we measured substantial amounts of IFN-β and– typical for epithelial cells– an even higher amount of IFN-λ (Figure 4A). IFN-α was hardly induced, possibly apart from the IRF5 KO, in which it reproducibly (in three out of three replicates) exceeded the lower limit of quantification (LLoQ) of the assay, and IFN-γ was not induced at all. Both, IFN-β and IFN-λ, were dependent on the presence of RIG-I and IRF3, but largely independent of any other IRF, including IRF1 and IRF2. Most likely due to positive feedback of the RLR system through IFNAR-/ISGF3-signaling, IRF9 KO had a slight impact on IFN-β production. Similar to IFN-α, there may further be a faint tendency towards increased IFN-β secretion in IRF5 KO (Figure 4A). This increase in IFN-β induction in IRF5 KO cells was further corroborated at the mRNA level upon transfection of a titration of 5’ppp-dsRNA (Supplementary Figure S6). For non-IFN cytokines (standard and alternative names, see Table 4), we observed an induction well above the LLoQ for IL-6 and TNF (Figure 4B), and to a smaller extent and/or close to the LLoQ for IL-10, IL-12p70, IL-2, IL-4, and IL-8 (Supplementary Figure S5A). Interestingly, while expression of TNF was independent of any IRF (predominantly driven by NF-κB), induction of IL-6 was fully dependent on IRF3. Albeit not robustly above the LLoQ, for IL-12p70 there was a tendency to be induced by RLR stimulation, and this was dependent on the presence of IRF1 (Supplementary Figure S5A). Production of chemokines is widely attributed to professional immune cells, however, also epithelial cells are capable of secreting certain chemokines upon viral infection [33]. In fact, we found massive induction of IP-10, MIP-1α, MIP-1β, and TARC, and to a lesser extent IL-8, MCP-4, and MDC (Figure 4C and Supplementary Figure S5B). Eotaxin and Eotaxin-3, as well as MCP-1, showed a tendency of being reduced upon RIG-I KO, too, albeit induction was less robust and either close to the LLoQ (Eotaxins) or above the upper limit (MCP-1) (Supplementary Figure S5B). Interestingly, there was a clear and virtually complete dependence on IRF3 for the expression of MIP-1α and MIP-1β, while IP-10 and TARC showed a substantial contribution of, but not complete dependence on IRF3. Similar to TNF (Figure 4B), secretion of IL-8, MCP-4, and MDC was dependent on RIG-I but independent of any IRF expression, indicating those chemokines may be largely induced by NF-κB (Figure 4C and Supplementary Figure S5B). Interestingly, at least under the chosen experimental conditions, feedback via type I/III IFN signaling (IRF9 KO) or via IRF7 was not essential for any of the produced cytokines/chemokines.

### 3.4. Replication of RNA and DNA Viruses under IRF KO Conditions

**Figure 5:**
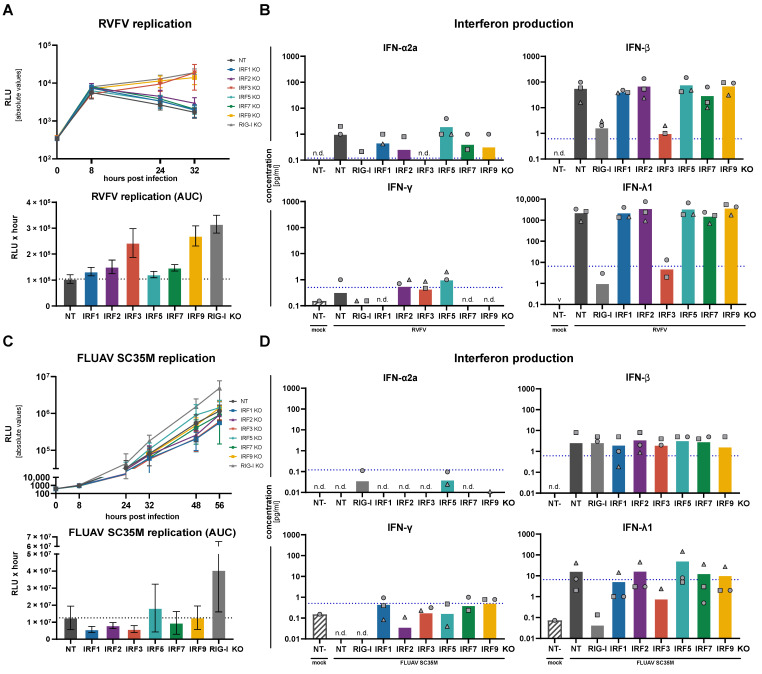
Impact of IRF KO on RNA virus replication and IFN production. A549^NT^, A549^IRF (as indicated) KO^, and A549^RIG-I KO^ cells were infected with Rift Valley fever virus (RVFV∆NSs-R-Luc) at an MOI of 0.01 (**A**,**B**), or with Influenza A Virus SC35M (FLUAV SC35M G-Luc) at an MOI of 0.001 (**C**,**D**). (**A**,**C**) Viral replication was assessed by measuring luciferase reporter activity at the indicated time points (top panels); the area under the curve (AUC) of the time courses is shown in the lower panels. Viral replication time-courses and AUC plots show mean ± SD of three independent experiments. (**B**,**D**) Supernatants of the infected cells were sampled at 32 h.p.i. (RVFV, **B**) or 48 h.p.i. (FLUAV, **D**) and analyzed for their IFN contents by an electroluminescent multiplex assay. Bars show means of three independent experiments with the three individual data points shown as symbols (same shapes indicate same experiment). “n.d.” indicates all measurements were non-detectable, “∨” indicates all measurements being below the cut-off of the Y-axis, and missing symbols indicate individual measurements below the cut-off or non-detectable. Dashed blue lines indicate the lower limit of quantification (LLoQ) for the respective analyte.

After having investigated the impact of the IRF KOs on numerous individual parameters of the antiviral response, we then aimed to understand how their overall effect would be on virus replication. While the IFN response is generally appreciated as a very broadly antiviral system, it has been described before that different classes of viruses are affected differently and are restricted by different subsets of ISGs and transcriptional responses [34,35]. Moreover, viruses have evolved elaborate strategies to counteract cell-intrinsic defenses at different levels. Therefore, we used a panel of luciferase reporter viruses from various classes to assess the impact of IRF KOs on their replication: Rift Valley fever virus (RVFV∆NSs, *phenuiviridae*/phlebovirus, (-)ssRNA); Influenza A virus (FLUAV, *orthomyxoviridae*, (-)ssRNA); Adenovirus type 5 (AdV5, *adnoviridae,* dsDNA); Herpes simplex virus 1 (HSV-1, *herpesviridae*, dsDNA). For each virus, we have measured viral replication kinetics in our panel of IRF KO cells and determined production of IFNs. RVFV robustly replicated and induced high amounts of both IFN-β and IFN-λ, while levels of IFN-α and IFN-γ were at or below the LLoQ (Figure 5A,B). KO of RIG-I almost completely abrogated IFN production, as did IRF3 KO. In line with this, viral replication was considerably rescued by these KOs, and additionally by IRF9 KO, which abrogates type I and III IFN signaling. Apart from a very slight tendency of the IRF2 KO to increase viral replication, none of the other IRFs affected RVFV replication (Figure 5A). Surprisingly, for FLUAV infection– at least with the chosen H7N7 (SC35M) reporter virus– the observed impact on viral replication was not only much smaller, but IRF3 as well as IRF1 and IRF2 KO slightly but reproducibly (across three replicates) dampened virus replication, while only RIG-I KO clearly rescued it. IRF5 also showed a tendency to rescue viral replication, while IRF7 and IRF9 KOs were largely not affecting FLUAV replication (Figure 5C). The overall small effect was also reflected in markedly lower IFN levels produced upon FLUAV infection as compared to RVFV (Figure 5D). We speculated this weak induction of cell-intrinsic antiviral pathways may be due to presence of the viral antagonistic protein NS1, however, also in an NS1 deletion version, we neither observed higher IFN induction nor a clearer impact of IRF KOs (not shown). Interestingly, and in contrast to the situation in RVFV infection, effects of IRF KO on FLUAV replication did not correlate with their effects on IFN production: IRF3 KO, as expected, reduced IFN-λ production substantially and comparably to RIG-I KO, nonetheless, it dampened virus replication. In contrast, IRF5 KO showed high IFN-β and, particularly, the highest IFN-λ levels among all KOs (albeit still at a very low level), but rather increased FLUAV replication (Figure 5C,D). This may indicate antiviral effects beyond the induction of IFN-signaling, particularly for IRF5. 

**Figure 6:**
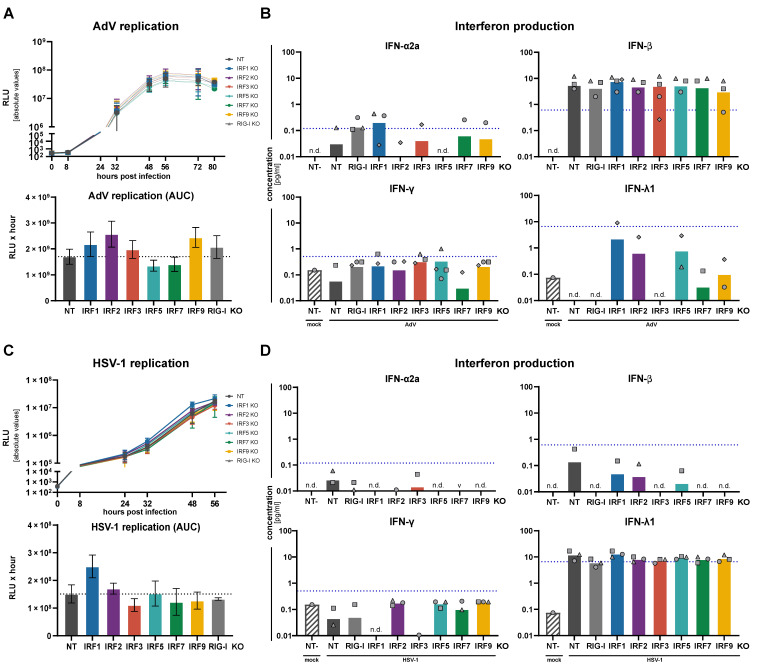
Impact of IRF KO on DNA virus replication and IFN production. A549^NT^, A549^IRF (as indicated) KO^, and A549^RIG-I KO^ cells were infected with Adenovirus (AdV, Ad5ΔfiberIL) at an MOI of 0.05 (**A**,**B**), or with Herpes simplex virus 1 (HSV-1, HSV1(17^+^)Lox-CheP2AGLuc) at an MOI of 0.1 (**C**,**D**). (**A**,**C**) Viral replication was assessed by measuring luciferase reporter activity at the indicated time points (top panels); the area under the curve (AUC) of the time courses is shown in the lower panels. Viral replication time-courses and AUC plots show mean ± SD of three independent experiments. (**B**,**D**) Supernatants of the infected cells were sampled at 56 h.p.i. (AdV, **B**) or 48 h.p.i. (HSV-1, **D**) and analyzed for their IFN contents by an electroluminescent multiplex assay. Bars show means of three independent experiments with the three individual data points shown as symbols (same shapes indicate same experiment). “n.d.” indicates all measurements were non-detectable, “∨” indicates all measurements being below the cut-off of the Y-axis, and missing symbols indicate individual measurements below the cut-off or non-detectable. Dashed blue lines indicate the lower limit of quantification (LLoQ) for the respective analyte.

We then turned to the DNA viruses, adenovirus (AdV) and HSV-1. Innate immune sensing of DNA viruses, in particular larger ones such as Herpesviruses, is complex and involves DNA as well as RNA sensors, including RIG-I [36]. Therefore, albeit not expected to be an optimal and complete positive control, for the sake of simplicity we stuck with RIG-I KO as the control in our panel of cells. Replication of both viruses was very efficient and not strongly affected by IRF KOs (Figure 6A,C). Adenovirus showed a certain tendency towards stronger replication upon IRF2 and IRF9 KO. Surprisingly, AdV infection led to low but detectable levels of IFN-β secretion, but no measurable IFN-λ, despite IFN-λ being the most prominent IFN in epithelial A549 cells upon any type of stimulation we have tested so far (Figure 6B). IFN-β was furthermore not dependent on the presence of IRF3, but–if anything–was only slightly reduced in IRF9 KO, possibly owing to a lack of positive feedback. These puzzling observations warrant further investigation in the future. In case of HSV-1, viral replication was somewhat boosted only in IRF1 KO, highlighting IRF1’s previously noted broadly antiviral potential [34]. All other IRFs were not affecting HSV-1 replication notably, including IRF3 (type I and III IFN production) and IRF9 (type I and III IFN signaling) (Figure 6C). As a striking mirror image to adenovirus, HSV-1 did trigger the production and secretion of IFN-λ, but almost completely prevented production of the type I IFN-β (Figure 6D, compare to Figure 6B). This most likely corresponds to the diverging strategies different viruses employ to tweak host responses to their own benefit, and/or may hint towards different sensing pathways or modalities. Actually, for both viruses, KO of individual IRFs did not noticeably affect IFN production, surprisingly not even the KO of IRF3, which was thought to be a major factor for the induction of type I and III IFN upon virus infection [37]. 

### 3.5. Impact of Increased Expression of IRF1, IRF5 and IRF7

This study aimed at investigating the role of individual IRFs in mounting cell-mediated antiviral response in a close to physiological setting. Hence, our focus was on functional KO instead of more artificial overexpression experiments. Nonetheless, for select IRFs, we further investigated the impact of their increased expression on IFN induction and, in particular, virus replication. For IRF1, it was previously reported that it possessed broadly antiviral functions, and it has also been implicated with the induction of type III IFN [34,38]. IRF5 has been initially described as being similar to IRF3 and IRF7, however, with differential specificity for IFN subtypes in human and murine systems [6,7,8]. It has been implicated in IFN induction downstream of Toll-like receptors, mainly TLR7 [4,5,39], and it furthermore showed a modest phenotype in several of our above-described experiments, which was largely reproducible across independent repetitions. Lastly, IRF7 is virtually absent at baseline in A549 cells (as is the case for almost all non-immune cells), but is strongly upregulated upon RLR stimulation. It is known as a major feedback regulator, and crucial for inducing IFN-α (see also Supplementary Figure S1C). We, hence, generated stably overexpressing cells by lentiviral transduction of IRF1, IRF5, and IRF7, as well as control cells stably transduced with the empty vector (Supplementary Figure S7).

**Figure 7:**
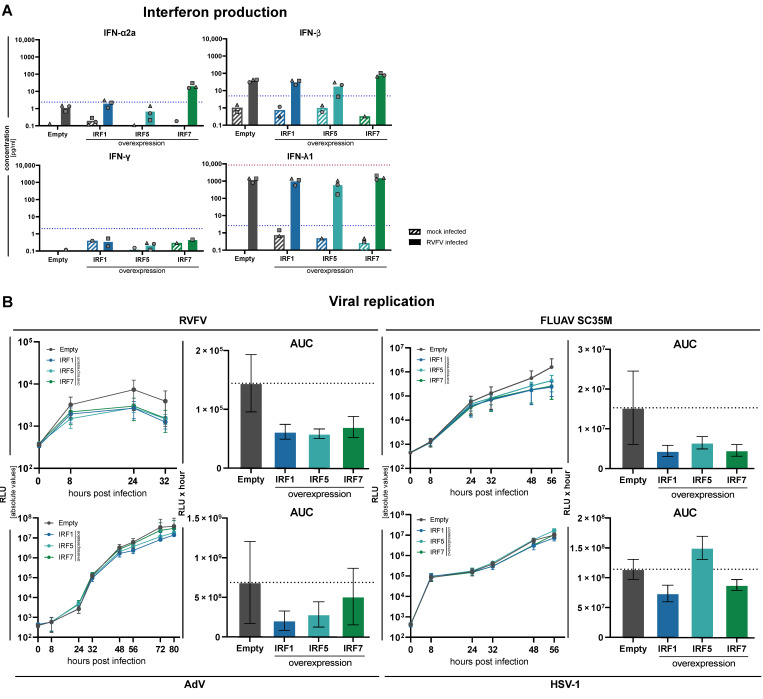
Impact of IRF1, -5 or -7 overexpression on IFN production and virus replication. (**A**) A549 cells stably transduced with an empty lentiviral expression vector or overexpressing IRF1, IRF5 or IRF7 were infected with RVFV (MOI 0.01) for 24 h and supernatants were analyzed for their IFN content by an electroluminescent multiplex assay. Dashed blue lines indicate the lower limit of quantification for the respective analyte. Bars show means of three independent experiments with the three individual data points shown as symbols (same shapes displays individual replicate). Missing symbols indicate individual measurements below the cut-off or non-detectable. (**B**) Empty vector or IRF1, -5 or -7 overexpressing cells were infected with the indicated viruses (RVFV, FLUAV, AdV, HSV-1) and viral replication was assessed by luciferase activity measurements at the indicated time points (left). The area under the curve (AUC) of the replication time courses is given as a bar chart (right). Viral replication time-courses and AUC plots show mean ± SD of three independent experiments.

First, we assessed the induction and secretion of IFNs in response to RLR stimulation in IRF overexpressing cells. Upon stimulation with RVFV, being the most potent inducer of IFNs among the tested viruses (Figure 5B), we measured type I (IFN-α2a, IFN-β), type II (IFN-γ), and type III (IFN-λ1) IFNs by an electroluminescence multiplex assay in the supernatant. In control cells, RVFV infection again led to the production of robust levels of IFN-β and very high levels of IFN-λ, and very low levels for IFN-α and INF-γ. The overexpression of IRF1 had little impact on the produced levels of IFNs, with only IFN-α being increased (Figure 7A). Notably, IFN-λ levels remained unchanged, although at an already very high level. Overexpression of IRF5, unexpectedly, led to a slight decrease in the produced levels of type I and III IFNs, while IRF7 overexpression increased IFN production across the range, with the strongest effects on IFN-α (Figure 7A). IRF7, hence, indeed acts as a positive feedback regulator and “diversifier” of RLR-mediated IFN production. 

We have observed a counterintuitive correlation between IFN production and the net effect on viral replication in IRF5 KO for Influenza virus, which may hint towards IFN independent roles of IRF5 in the antiviral defense discussed earlier (Figure 5C,D). We, therefore, infected IRF1, 5, and 7 overexpressing cells with the four luciferase reporter viruses and recorded viral replication over time, analogously to the experiments in KO conditions (Figure 6 and Figure 7). In fact, we found a profound impact of the overexpression of all three IRFs on the two RNA viruses RVFV and FLUAV (Figure 7B), including IRF5, which reduced rather than increased IFN production (Figure 7A). While this is surprising, it fits the observation of FLUAV replication in IRF5 KO, in which both virus replication and IFN production were slightly increased (Figure 5C,D), as well as to the increased IFN-β mRNA levels upon transfection of 5’ppp-dsRNA in IRF5 KO cells (Supplemenatary Figure S6). As for the two tested DNA viruses, HSV-1 was less affected by overexpression of the IRFs; only IRF1 and IRF7 decreased replication at 48 h post infection, with IRF7 catching up with the empty vector controls 56 h post infection (Figure 7E). The slight but continuous impact of IRF1 mirrors the observed increase in replication upon IRF1 KO (Figure 6C). Interestingly, for adenovirus we observed a notable and reproducible reduction in replication upon IRF1 and IRF5 overexpression (Figure 7B), potentially underscoring IFN independent mechanisms of antiviral activity for these two transcription factors. 

## 4. Discussion

**Figure 8:**
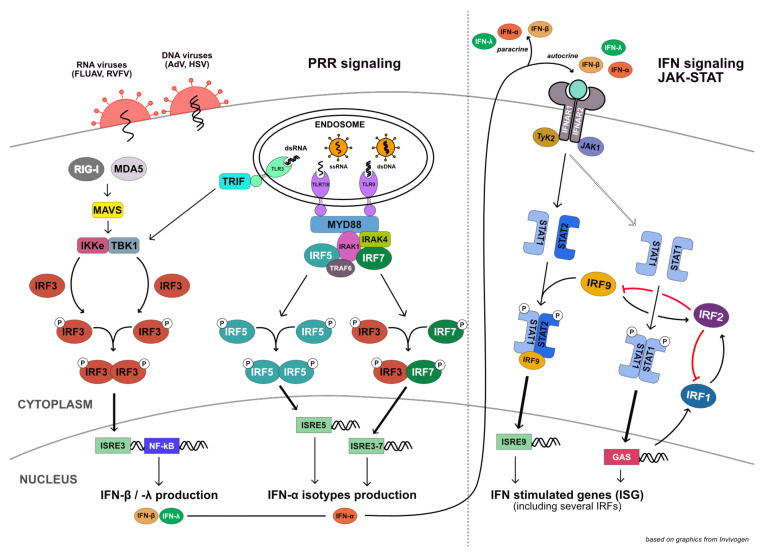
Schematic overview of the most prominent cell-intrinsic antiviral signaling pathways. The involvement of well-characterized IRF transcription factors is indicated, but does not reflect the actual complexity with various IRFs (e.g., IRF7, IRF1) only being expressed after initial stimulation of the pathway and ensuing positive transcriptional feedback. The figure is based on an illustration by Invivogen (https://www.invivogen.com/review-type1-ifn-production (accessed on 1 September 2021)).

The IRF family of transcription factors is well known for their crucial involvement in the transcriptional regulation of innate immune response programs, particularly in the antiviral defense system. They have an intricate relationship with the IFN system, critically mediating and regulating both IFN induction downstream of pathogen recognition receptors, as well as signaling downstream of all three types of IFNs and their respective receptors (see Figure 8 for reference; comprehensively reviewed in [10]). Beyond the antiviral or antimicrobial response system, over the last decade various other functions of IRFs have been described, establishing roles in general cellular homeostasis and in particular in regulating cell proliferation, cell death pathways, and in controlling oncogenesis [40]. In our present study, we did not investigate effects on cellular homeostasis, however, the panel of IRF KO cell clones created may very well serve future studies on such cell-biological aspects. Instead, we specifically focused on the IRFs’ role in the RLR-mediated antiviral defense system of non-immune cells. In contrast to a large body of work available in literature on individual IRFs’ functions and molecular mechanisms [41,42], we aimed to establish their relative functional contribution to the mounting of an IFN response upon RLR (specifically RIG-I) stimulation and upon infection with RNA or DNA viruses.

Downstream of virus-detecting PRRs, IRF3 and IRF7, have been reported to be the central transcription factors driving the expression of antiviral genes and, in particular, of type I and III IFNs [10,43]. While IRF3 is constitutively expressed across almost all cell types, high basal expression of IRF7 is largely restricted to immune cells, such as B-cells and, in particular, plasmacytoid dendritic cells [43]. In line with this, we found IRF3 robustly expressed in our A549 cells with no further increase upon IFN stimulation. In contrast, IRF7 was virtually absent, but strongly induced by IFN treatment and virus infection, underscoring its role as a positive feedback mechanism of the antiviral system. Accordingly, KO of IRF3 virtually completely blocked the induction of type I and III IFNs and IFN-induced ISGs, including IRF1, IRF2, IRF9, and particularly pronounced IRF7. It also strongly reduced or fully abrogated some proinflammatory cytokines, such as RANTES, IL-6, IP-10, MIP-1α/β, and TARC, while IRF3 was dispensable for other, putatively NF-κB-induced factors such as TNFAIP3 (A20), TNF, MCP-4, and MDC. Previous studies mostly investigated the transcriptional profile of IRF3 in the context of immune cells [44], but here we confirm that several chemokines are also substantially induced by IRF3 in epithelial cells. As for the overall impact on the functional antiviral response, RVFV ∆NSs, the virus most potently stimulating IFN production, replicated considerably better in the absence of IRF3, comparable to conditions lacking the sensor RIG-I. IRF7, on the other hand, was dispensable in virtually all experimental settings; most likely, this was due to the high basal expression of IRF3. Only production of IFN-α, a cytokine not induced by IRF3 [43], showed a certain reduction upon KO of IRF7, albeit its levels were already close to the lower limit of quantification in control cells. Reciprocally, overexpression of IRF7 considerably increased IFN-α production upon RVFV infection and, correspondingly, impacted viral replication, especially upon RVFV and FLUAV infection (Figure 7).

The IFNs produced upon PRR stimulation are secreted and trigger their cognate receptors (IFNAR for type I and IFNLR for type III IFNs). IFN receptor downstream signaling involves the phosphorylation and activation of STAT proteins, most importantly STAT1 and STAT2 [45]. Canonically, STAT1:STAT2 heterodimers form and associate with IRF9 to form the trimeric transcription factor ISGF3; recently, it was suggested that complexes of STAT2 and IRF9 form a constitutive transcription factor under homeostatic conditions, and upon IFN signaling, STAT1 only associates in the nucleus [46]. Hence, it may be that the here employed constitutive KO of IRF9 influences the homeostatic expression of several genes. However, we have not observed an impact on background IFIT1 or MX1 expression (Figure 2) and no marked effect on early kinetics (<8 h) of RLR signaling (Figure 3). In contrast, we did observe a strong impact of IRF9 KO on late events, such as IFIT1 expression later upon RLR stimulation or upon IFN treatment. Additionally, IFN-inducible IRFs IRF7 and IRF2 were strongly affected, albeit IRF2 was only induced upon RLR stimulation in our hands (Supplementary Figure S2, discussed below). In contrast, IRF1 induction was not at all reduced by IRF9 KO, underscoring its dependence on STAT1 homodimers instead of ISGF3. Strikingly, in the absence of IRF9, induction of IRF1 was even considerably increased upon type I IFN treatment (Figure 1), suggesting a forced lack of ISGF3 formation leads to an increase in STAT1 homodimers, potentially shifting the classical type I IFN response more towards a type II-like response pattern. The fact this was only observed in the IFN treatment condition, but not so in the RVFV infection (Supplementary Figure S2)—despite an overall comparably strong response amplitude (e.g., compare IRF7 or IRF9 induction)– may be connected to the types of IFN involved. While the exogenously applied IFN was type I only (IFN-α), RVFV infection leads to a type III dominated IFN induction (Figure 6A and Figure 8A), and a recent study demonstrated IRF1 induction to be profoundly stronger downstream of type I rather than type III IFN signaling [17]. Still, this is somewhat at odds with our observation of a markedly stronger overall expression of IRF1 upon RVFV infection, which may be due to complex feedback mechanisms. On the induction of other cytokines in response to RLR stimulation, IRF9 KO had little to no impact, with the caveat that replicates tended to vary more for IRF9, particularly for analytes close to the quantification limit (Figure 4, Supplementary Figure S5). Not surprisingly, IRF9 KO clearly rescued replication of RVFV, which induced high amounts of IFN, and possibly slightly also replication of adenovirus (Figure 6A).

The two induced and allegedly antagonistic IRFs [18,47], IRF1 and IRF2, in our experimental system turned out to be of less profound relevance than expected from the literature [15,48]. Although reported differently, we could not find mutual regulation of their expression, neither upon IFN treatment nor RVFV infection. IRF2 surprisingly did not affect the expression of any of the measured ISGF3 dependent ISGs, neither of IRF7, IRF9, IFIT1, nor MX1. As for the other cytokines, only IL-12p70 appeared to be reciprocally regulated by IRF1 and IRF2, however, absolute levels were close to the LLoQ even in the control cells (Supplementary Figure S5). In line with the minute effects of IRF2 KO across all our read-outs, we also detected no notable impact on the replication of any of the tested viruses. These results may indicate that the reported transcriptional repression by IRF2 [18] may be weaker in A549 cells or the conditions of stimulation employed. Interestingly, although we have recently confirmed that type I IFN does induce IRF2 expression [14], we did not observe this in the current study (Figure 1). Besides the different cell lines used in the two studies, we here employed relatively low amounts of IFN-α (200 IU/mL) and measured IRF2 expression 16 h post-treatment. Previously, we found IRF2 to be induced very transiently and most strongly at early times post-treatment. It is conceivable, that we would be able to detect IRF2 expression at earlier time points in A549 cells, too; indeed, at protein level, there is a slight tendency of increased IRF2 levels, possibly owing to the longer half-life of protein as compared to mRNA (Figure 1). Whereas this may indicate that repressive functions may also be temporally restricted, even in our dynamic assessment, IRF2 KO did not strongly increase IFIT1 expression at the early time points (Supplementary Figure S4). 

For IRF1 KO, we similarly only found subtle, if any, impact across the range of our experiments. Notably, expression of no other IRF seemed to be affected by the absence of IRF1, neither basal nor induced levels. Fitting to its described role in maintaining homeostatic expression of antiviral factors [49], background levels of IFIT1 and MX1 appeared to be slightly reduced in IRF1 KO (as well as in IRF2 KO). However, no impact was observed under stimulated conditions (Figure 2, Supplementary Figure S3). Accordingly, there was also minimal impact on virus replication. Notably, only HSV-1 replication seemed to benefit from IRF1 KO, whereas FLUAV replication was even dampened (Figure 5C and Figure 7C). In both cases, the effects did not correlate to type I or III IFN levels, which were largely unaffected by IRF1 KO. Because of these slight indications in our own data, but particularly given the strong antiviral effect of IRF1 in previous studies [15,48], we complemented our KO experiments with IRF1 overexpression, also in A549 cells. While IRF1 again showed no notable effect on IFN-β or -λ induction upon virus (RVFV) infection and only minimally increased IFN-α, its overexpression inhibited the replication of all four tested viruses (Figure 7B), underscoring its capacity to induce direct antiviral effects. This is in line with the findings by Schoggins and colleagues, in whose ISG screens IRF1 repeatedly emerged as one of the most potent factors against a variety of virus families when singly expressed [48]. As for the magnitude of the effect, which only was around 2- to 4-fold in our experiments, it needs to be kept in mind that we used stably transduced cells, which may exhibit significantly lower steady-state amounts of IRF1 than transiently transfected/transduced cells used in other studies. Overall, understanding the relative importance of IRF1 to acute antiviral defense was one of the central interests for us in this work. Somewhat to our surprise, the contribution of IRF1, in general, was very modest and largely dispensable. To us, this indicates that the role of this factor likely lies in regulatory functions beyond the classical IFN/ISG response and may instead rather regulate cell homeostasis, cell survival, and (induced) apoptosis in response to viral infection and other molecular cues [40,50]. It may further be important in other cell types, most importantly professional immune cells. For example, IRF1 and IRF2 have been described to play a crucial role in macrophage polarization [51]. Such cell-biological and immunological effects were not covered by the assays in our present study, hence, our current findings do not at all argue against an important role for IRF1 in innate immunity.

Lastly, we have studied the role of IRF5. It may be best known for its function downstream of TLR7, -8, and -9, where it reportedly takes up the role of IRF3 and -7 in other PRR pathways [4]. Very recently, TASL has been identified as the specific adaptor molecule (complementing the adaptors TRIF, MAVS, and STING) linking TLR7/8/9 to the antiviral transcriptional program [5]. It was, hence, known that IRF5 can induce a similar transcriptional response to IRF3 and -7, trans-activating type I and III IFN genes. IRF5 has also gained some attention in recent years for its involvement in autoinflammatory and autoimmune diseases [52], and much like IRF1, it has been implicated in induced cell death [53]. While such cell-biological properties, again, could not be assessed in our current study, we did find a slight but coherent trend of effects of IRF5 in the RLR/IFN response system. Upon KO of IRF5, across many different experiments we observed a slight increase in the levels of, particularly type I, IFNs (e.g., IFN-β, Supplementary Figure S6) and ISGs (e.g., IRF7, Figure 1). This was mirrored by overexpression experiments, in which elevated IRF5 levels rather decreased the induction of type I and III IFNs upon RLR stimulation by RVFV infection (Figure 7). Despite being of very modest strength, these findings are surprising, and may suggest presence of an antagonism between IRF3 (and possibly -7) and IRF5. While this, to our knowledge, has not been reported before, a non-trivial interaction and cooperation between IRF3, -7, and -5 downstream of MAVS has been observed in flavivirus infection of dendritic cells [9]. Furthermore, despite rather reducing the levels of IFN induction, IRF5 appeared to be antiviral. Upon overexpression, we observed reduced replication of RVFV, FLUAV, and AdV (Figure 7). Vice versa, KO of IRF5 led to a slight increase in FLUAV replication, in astonishing contrast to all other IRF KOs (Figure 5C). This suggests that IRF5 may have additional, potentially cell-biological [52], effects not covered by our read-outs, that may mediate IFN-independent antiviral efficacy against at least some viruses. Although effect sizes were too small in our experiments, and our model systems too limited to draw general conclusions, these findings invite future studies on the specific role of IRF5 in the RLR-mediated IFN response (possible antagonism to IRF3/7) and potential direct, IFN-independent antiviral effects. 

In conclusion, we here presented– to our knowledge– the first systematic side-by-side comparison of the functional impact of IRF1, -2, -3, -5, -7, and -9 in virus infection, RLR stimulation, and IFN treatment in epithelial cells. We confirmed the vital importance of IRF3 (IFN induction phase) and IRF9 (IFN effector phase) in these responses and found that the contributions of other IRFs were relatively mild. This was true even for IRF1, which was reported to possess strong antiviral potency, and IRF2, which has been implicated in repressing IFN-triggered transcriptional responses. Interestingly, IRF5 exhibited small but consistent effects in KO as well as overexpression, possibly suggesting a previously unrecognized role in suppressing IRF3-mediated IFN induction and inviting future investigation. While our study focused only on one cell type, one PRR system (RLRs), and (largely) only on known antiviral functions (e.g., cytokine induction), it may serve as a profound basis for future investigations into various directions.

## Figures and Tables

**Table 1:** Target-specific guide RNAs used for cloning into lentiCRISPR v2 plasmid.

Name	Forward Sequence (5′-3′)
NT	gtattactgatattggt
IFNAR1	gaccctagtgctcgtcgccg
IFNGR1	cctggtgctcacccagacgg
IFNLR	caccggagtaccagatcatgccac
IRF1	tggtgagaggtggaagcatc
IRF2	gataaactccaacacgatcc
IRF3	cggaaattcctcttccaggt
IRF5	gccagggcttcagccgcacg
IRF7	atcagcagcggctgctatga
IRF9	gagggagtcctggagcacag
RELA	gctcaatgatctccacatag
RIG-I	tggagctccaggaggaaggc

**Table 2:** Primary Antibodies used for Immunoblotting (1:1000 dilution).

Name	Identifier
mouse monoclonal anti-β-Actin	A5441, RRID: AB_476744
rabbit polyclonal anti-Calnexin	ADI-SPA-865-F, RRID: AB_11180747
rabbit monoclonal anti-IRF1 (D5E4) XP	*8478*, RRID: AB_10949108
rabbit monoclonal anti-IRF2 [EPR4644(2)]	ab124744, RRID: AB_10974405
rabbit monoclonal anti-IRF3 (D6I4C) XP	11904, RRID: AB_2722521
rabbit polyclonal anti-IRF5	HPA046700, RRID: AB_2679761
rabbit polyclonal anti-IRF7	4920, RRID: AB_2127551
mouse monoclonal anti-IRF9 Clone 6/ISGF3γ	610285, RRID: AB_397680
mouse monoclonal anti-RIG-I (Alme-1)	AG-20B-0009, RRID: AB_2490189
mouse monoclonal anti-NFkB p65 (L8F6)	6956, RRID: AB_10828935
Mouse monoclonal anti-HA (Clone HA-7)	H3663, RRID: AB_262051

**Table 3:** Gene specific primers used for qRT-PCR.

Name	Forward Sequence (5′-3′)	Reverse Sequence (5′-3′)
GAPDH	tcggagtcaacggatttggt	ttcccgttctcagccttgac
IFIT1	gaatagccagatctcagaggagc	ccatttgtactcatggttgctgt
IFNB1	*cgccgcattgaccatcta*	gacattagccaggaggttctc
IRF1	cctgactccagcactgtcg	tgggtgacacctggaagttg
IRF2	acggtgaacatcatagtttagga	ggggagatctgcagagggta
IRF3	tgggcccccagatctgatta	gcacaaccttgaccatcacg
IRF5	aagatctacgaggtctgctccaat	atcctctgcagctcttcctctt
IRF7	ttggctcctgagagggca	cagcccaggccttgaagatg
IRF9	tcctccagagccagactact	caatccaggctttgcacctg
MX1	accattccaaggaggtgcag	tgcgatgtccacttcggaaa
RANTES	gctgtcatcctcattgctactg	tggtgtagaaatactccttgatgtg
TNFAIP3 (A20)	tcctcaggctttgtatttgagc	tgtgtatcggtgcatggtttta

**Table 4:** Cytokine/chemokine nomenclature. As for many cytokines and chemokines commonly used names still exist besides their current systematic nomenclature, we decided to give the common name in the text and provide a table with alternative and systematic names.

Common Name	Alternative Name(s)	Systematic Name
MCP-1	MCAF, …	CCL2
MIP-1α	--	CCL3
MIP-1β	--	CCL4
RANTES	--	CCL5
Eotaxin	--	CCL11
MCP-4	NCC-1, Ckβ10, …	CCL13
TARC	dendrokine, ABCD-2	CCL17
MDC	DC/β-CK, ABCD-1, …	CCL22
Eotaxin-3	MIP-4α, IMAC, TSC-1	CCL26
IL-8	NAP-1, MDNCF, GCP-1, …	CXCL8
IP-10	CRG-10, IFI10, …	CXCL10

## Data Availability

With regard to maximal data transparency, we attempted to give as much raw data as possible throughout our manuscript, showing individual replicate measurements rather than only mean values. All data (including not shown replicate Western blots, etc.) are available at request from the corresponding author.

## Supplementary Materials

The following are available online at https://www.mdpi.com/article/10.3390/cells10102600/s1, Supplementary Figure S1: Validation of A549 Knockout Clones, Supplementary Figure S2: Knockout validation and mutual dependency of IRFs (RVFV), Supplementary Figure S3: Impact of IRFs on ISG induction, Supplementary Figure S4: Impact of IRFs on RIG-I-dependent and RIG-I-independent signaling, Supplementary Figure S5: Proinflammatory cytokine and chemokine profiles of IRF KO cell lines after RIG-I-specific stimulus, Supplementary Figure S6: IFN-β production upon IRF5 KO, Supplementary Figure S7: Validation of A549 IRF1, IRF5 and IRF7 overexpression cell lines. Statistics.xlsx: results of statistical testing for Figure 4, Figure 5, Figure 6 and Figure 7 (see methods).

## References

[1] Bastard P., Rosen L.B., Zhang Q., Michailidis E., Hoffmann H.H., Zhang Y., Dorgham K., Philippot Q., Rosain J., Beziat V. (2020). Autoantibodies against type I IFNs in patients with life-threatening COVID-19. Science.

[2] Zhang X., Tan Y., Ling Y., Lu G., Liu F., Yi Z., Jia X., Wu M., Shi B., Xu S. (2020). Viral and host factors related to the clinical outcome of COVID-19. Nature.

[3] Loske J., Rohmel J., Lukassen S., Stricker S., Magalhaes V.G., Liebig J., Chua R.L., Thurmann L., Messingschlager M., Seegebarth A. (2021). Pre-activated antiviral innate immunity in the upper airways controls early SARS-CoV-2 infection in children. Nat. Biotechnol..

[4] Schoenemeyer A., Barnes B.J., Mancl M.E., Latz E., Goutagny N., Pitha P.M., Fitzgerald K.A., Golenbock D.T. (2005). The interferon regulatory factor, IRF5, is a central mediator of toll-like receptor 7 signaling. J. Biol. Chem..

[5] Heinz L.X., Lee J., Kapoor U., Kartnig F., Sedlyarov V., Papakostas K., Cesar-Razquin A., Essletzbichler P., Goldmann U., Stefanovic A. (2020). TASL is the SLC15A4-associated adaptor for IRF5 activation by TLR7-9. Nature.

[6] Barnes B.J., Moore P.A., Pitha P.M. (2001). Virus-specific activation of a novel interferon regulatory factor, IRF-5, results in the induction of distinct interferon alpha genes. J. Biol. Chem..

[7] Yanai H., Chen H.M., Inuzuka T., Kondo S., Mak T.W., Takaoka A., Honda K., Taniguchi T. (2007). Role of IFN regulatory factor 5 transcription factor in antiviral immunity and tumor suppression. Proc. Natl. Acad. Sci. USA.

[8] Paun A., Reinert J.T., Jiang Z., Medin C., Balkhi M.Y., Fitzgerald K.A., Pitha P.M. (2008). Functional characterization of murine interferon regulatory factor 5 (IRF-5) and its role in the innate antiviral response. J. Biol. Chem..

[9] Lazear H.M., Lancaster A., Wilkins C., Suthar M.S., Huang A., Vick S.C., Clepper L., Thackray L., Brassil M.M., Virgin H.W. (2013). IRF-3, IRF-5, and IRF-7 coordinately regulate the type I IFN response in myeloid dendritic cells downstream of MAVS signaling. PLoS Pathog..

[10] Negishi H., Taniguchi T., Yanai H. (2018). The Interferon (IFN) Class of Cytokines and the IFN Regulatory Factor (IRF) Transcription Factor Family. Cold Spring Harb. Perspect. Biol..

[11] Bluyssen A.R., Durbin J.E., Levy D.E. (1996). ISGF3 gamma p48, a specificity switch for interferon activated transcription factors. Cytokine Growth Factor Rev..

[12] Hida S., Ogasawara K., Sato K., Abe M., Takayanagi H., Yokochi T., Sato T., Hirose S., Shirai T., Taki S. (2000). CD8(+) T cell-mediated skin disease in mice lacking IRF-2, the transcriptional attenuator of interferon-alpha/beta signaling. Immunity.

[13] Decker T., Muller M., Stockinger S. (2005). The yin and yang of type I interferon activity in bacterial infection. Nat. Rev. Immunol..

[14] Kok F., Rosenblatt M., Teusel M., Nizharadze T., Magalhaes V.G., Dachert C., Maiwald T., Vlasov A., Wasch M., Tyufekchieva S. (2020). Disentangling molecular mechanisms regulating sensitization of interferon alpha signal transduction. Mol. Syst. Biol..

[15] Feng H., Zhang Y.B., Gui J.F., Lemon S.M., Yamane D. (2021). Interferon regulatory factor 1 (IRF1) and anti-pathogen innate immune responses. PLoS Pathog..

[16] Kimura T., Nakayama K., Penninger J., Kitagawa M., Harada H., Matsuyama T., Tanaka N., Kamijo R., Vilcek J., Mak T.W. (1994). Involvement of the IRF-1 transcription factor in antiviral responses to interferons. Science.

[17] Forero A., Ozarkar S., Li H., Lee C.H., Hemann E.A., Nadjsombati M.S., Hendricks M.R., So L., Green R., Roy C.N. (2019). Differential Activation of the Transcription Factor IRF1 Underlies the Distinct Immune Responses Elicited by Type I and Type III Interferons. Immunity.

[18] Harada H., Fujita T., Miyamoto M., Kimura Y., Maruyama M., Furia A., Miyata T., Taniguchi T. (1989). Structurally similar but functionally distinct factors, IRF-1 and IRF-2, bind to the same regulatory elements of IFN and IFN-inducible genes. Cell.

[19] Willemsen J., Wicht O., Wolanski J.C., Baur N., Bastian S., Haas D.A., Matula P., Knapp B., Meyniel-Schicklin L., Wang C. (2017). Phosphorylation-Dependent Feedback Inhibition of RIG-I by DAPK1 Identified by Kinome-wide siRNA Screening. Mol. Cell.

[20] Krischuns T., Gunl F., Henschel L., Binder M., Willemsen J., Schloer S., Rescher U., Gerlt V., Zimmer G., Nordhoff C. (2018). Phosphorylation of TRIM28 Enhances the Expression of IFN-beta and Proinflammatory Cytokines During HPAIV Infection of Human Lung Epithelial Cells. Front. Immunol..

[21] Urban C., Welsch H., Heine K., Wust S., Haas D.A., Dachert C., Pandey A., Pichlmair A., Binder M. (2020). Persistent Innate Immune Stimulation Results in IRF3-Mediated but Caspase-Independent Cytostasis. Viruses.

[22] Cadena C., Ahmad S., Xavier A., Willemsen J., Park S., Park J.W., Oh S.W., Fujita T., Hou F., Binder M. (2019). Ubiquitin-Dependent and -Independent Roles of E3 Ligase RIPLET in Innate Immunity. Cell.

[23] Heigwer F., Kerr G., Boutros M. (2014). E-CRISP: Fast CRISPR target site identification. Nat. Methods.

[24] Sanjana N.E., Shalem O., Zhang F. (2014). Improved vectors and genome-wide libraries for CRISPR screening. Nat. Methods.

[25] Robinson J.T., Thorvaldsdottir H., Winckler W., Guttman M., Lander E.S., Getz G., Mesirov J.P. (2011). Integrative genomics viewer. Nat. Biotechnol..

[26] (2016). The ORFeome Collaboration: A genome-scale human ORF-clone resource. Nat. Methods.

[27] Katzen F. (2007). Gateway((R)) recombinational cloning: A biological operating system. Expert Opin. Drug Discov..

[28] Reuther P., Gopfert K., Dudek A.H., Heiner M., Herold S., Schwemmle M. (2015). Generation of a variety of stable Influenza A reporter viruses by genetic engineering of the NS gene segment. Sci. Rep..

[29] Habjan M., Penski N., Spiegel M., Weber F. (2008). T7 RNA polymerase-dependent and -independent systems for cDNA-based rescue of Rift Valley fever virus. J. Gen. Virol..

[30] Rivera A.A., Wang M., Suzuki K., Uil T.G., Krasnykh V., Curiel D.T., Nettelbeck D.M. (2004). Mode of transgene expression after fusion to early or late viral genes of a conditionally replicating adenovirus via an optimized internal ribosome entry site in vitro and in vivo. Virology.

[31] Devadas D., Koithan T., Diestel R., Prank U., Sodeik B., Dohner K. (2014). Herpes simplex virus internalization into epithelial cells requires Na+/H+ exchangers and p21-activated kinases but neither clathrin- nor caveolin-mediated endocytosis. J. Virol..

[32] Ye J., Coulouris G., Zaretskaya I., Cutcutache I., Rozen S., Madden T.L. (2012). Primer-BLAST: A tool to design target-specific primers for polymerase chain reaction. BMC Bioinform..

[33] Li K., Li N.L., Wei D., Pfeffer S.R., Fan M., Pfeffer L.M. (2012). Activation of chemokine and inflammatory cytokine response in hepatitis C virus-infected hepatocytes depends on Toll-like receptor 3 sensing of hepatitis C virus double-stranded RNA intermediates. Hepatology.

[34] Schoggins J.W., Rice C.M. (2011). Interferon-stimulated genes and their antiviral effector functions. Curr. Opin. Virol..

[35] Seifert L.L., Si C., Saha D., Sadic M., de Vries M., Ballentine S., Briley A., Wang G., Valero-Jimenez A.M., Mohamed A. (2019). The ETS transcription factor ELF1 regulates a broadly antiviral program distinct from the type I interferon response. PLoS Pathog..

[36] Zhao J., Qin C., Liu Y., Rao Y., Feng P. (2020). Herpes Simplex Virus and Pattern Recognition Receptors: An Arms Race. Front. Immunol..

[37] Odendall C., Kagan J.C. (2015). The unique regulation and functions of type III interferons in antiviral immunity. Curr. Opin. Virol..

[38] Odendall C., Dixit E., Stavru F., Bierne H., Franz K.M., Durbin A.F., Boulant S., Gehrke L., Cossart P., Kagan J.C. (2014). Diverse intracellular pathogens activate type III interferon expression from peroxisomes. Nat. Immunol..

[39] Takaoka A., Yanai H., Kondo S., Duncan G., Negishi H., Mizutani T., Kano S., Honda K., Ohba Y., Mak T.W. (2005). Integral role of IRF-5 in the gene induction programme activated by Toll-like receptors. Nature.

[40] Yanai H., Negishi H., Taniguchi T. (2012). The IRF family of transcription factors: Inception, impact and implications in oncogenesis. Oncoimmunology.

[41] Mancino A., Natoli G. (2016). Specificity and Function of IRF Family Transcription Factors: Insights from Genomics. J. Interferon Cytokine Res..

[42] Tamura T., Yanai H., Savitsky D., Taniguchi T. (2008). The IRF family transcription factors in immunity and oncogenesis. Annu. Rev. Immunol..

[43] Hiscott J. (2007). Triggering the innate antiviral response through IRF-3 activation. J. Biol. Chem..

[44] Ourthiague D.R., Birnbaum H., Ortenlof N., Vargas J.D., Wollman R., Hoffmann A. (2015). Limited specificity of IRF3 and ISGF3 in the transcriptional innate-immune response to double-stranded RNA. J. Leukoc. Biol..

[45] Stetson D.B., Medzhitov R. (2006). Type I interferons in host defense. Immunity.

[46] Platanitis E., Demiroz D., Schneller A., Fischer K., Capelle C., Hartl M., Gossenreiter T., Muller M., Novatchkova M., Decker T. (2019). A molecular switch from STAT2-IRF9 to ISGF3 underlies interferon-induced gene transcription. Nat. Commun..

[47] Yan Y., Zheng L., Du Q., Yan B., Geller D.A. (2020). Interferon regulatory factor 1 (IRF-1) and IRF-2 regulate PD-L1 expression in hepatocellular carcinoma (HCC) cells. Cancer Immunol. Immunother..

[48] Schoggins J.W., Wilson S.J., Panis M., Murphy M.Y., Jones C.T., Bieniasz P., Rice C.M. (2011). A diverse range of gene products are effectors of the type I interferon antiviral response. Nature.

[49] Panda D., Gjinaj E., Bachu M., Squire E., Novatt H., Ozato K., Rabin R.L. (2019). IRF1 Maintains Optimal Constitutive Expression of Antiviral Genes and Regulates the Early Antiviral Response. Front. Immunol..

[50] Kuriakose T., Zheng M., Neale G., Kanneganti T.D. (2018). IRF1 Is a Transcriptional Regulator of ZBP1 Promoting NLRP3 Inflammasome Activation and Cell Death during Influenza Virus Infection. J. Immunol..

[51] Chistiakov D.A., Myasoedova V.A., Revin V.V., Orekhov A.N., Bobryshev Y.V. (2018). The impact of interferon-regulatory factors to macrophage differentiation and polarization into M1 and M2. Immunobiology.

[52] Kaur A., Lee L.H., Chow S.C., Fang C.M. (2018). IRF5-mediated immune responses and its implications in immunological disorders. Int. Rev. Immunol..

[53] Hu G., Barnes B.J. (2009). IRF-5 is a mediator of the death receptor-induced apoptotic signaling pathway. J. Biol. Chem..

