# Supplementary material for: Comparative Analysis of Six IRF Family Members in Alveolar Epithelial Cell-Intrinsic Antiviral Responses
Source: Cells. 2021 Sep 30;10(10):2600. doi: 10.3390/cells10102600 (PMC8533862; doi:10.3390/cells10102600)
Supplement: Supplementary file 1 [file cells-10-02600-s001.zip › cells-1342426-supplementary/Supplementary Figures.pdf]

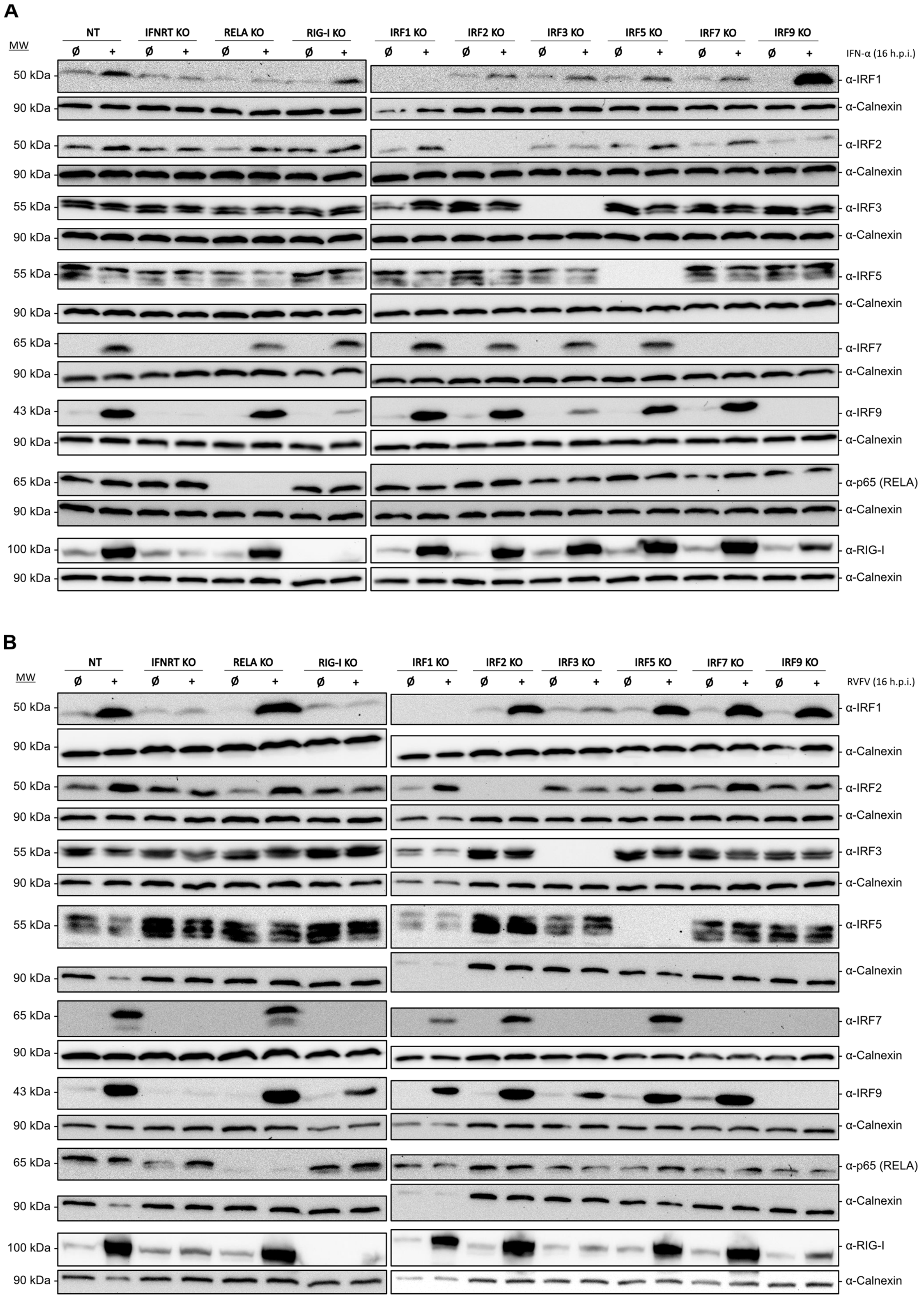
A
NT
IFNRT KO
RELA KO
RIG-I KO
IRF1 KO
IRF2 KO
IRF3 KO
IRF5 KO
IRF7 KO
IRF9 KO
MW
Ø
+
IFN-α (16 h.p.i.)
50 kDa
90 kDa
55 kDa
65 kDa
43 kDa
100 kDa
α-IRF1
α-Calnexin
α-IRF2
α-IRF3
α-IRF5
α-IRF7
α-IRF9
α-p65 (RELA)
α-RIG-I
B
RVFV (16 h.p.i.)

C

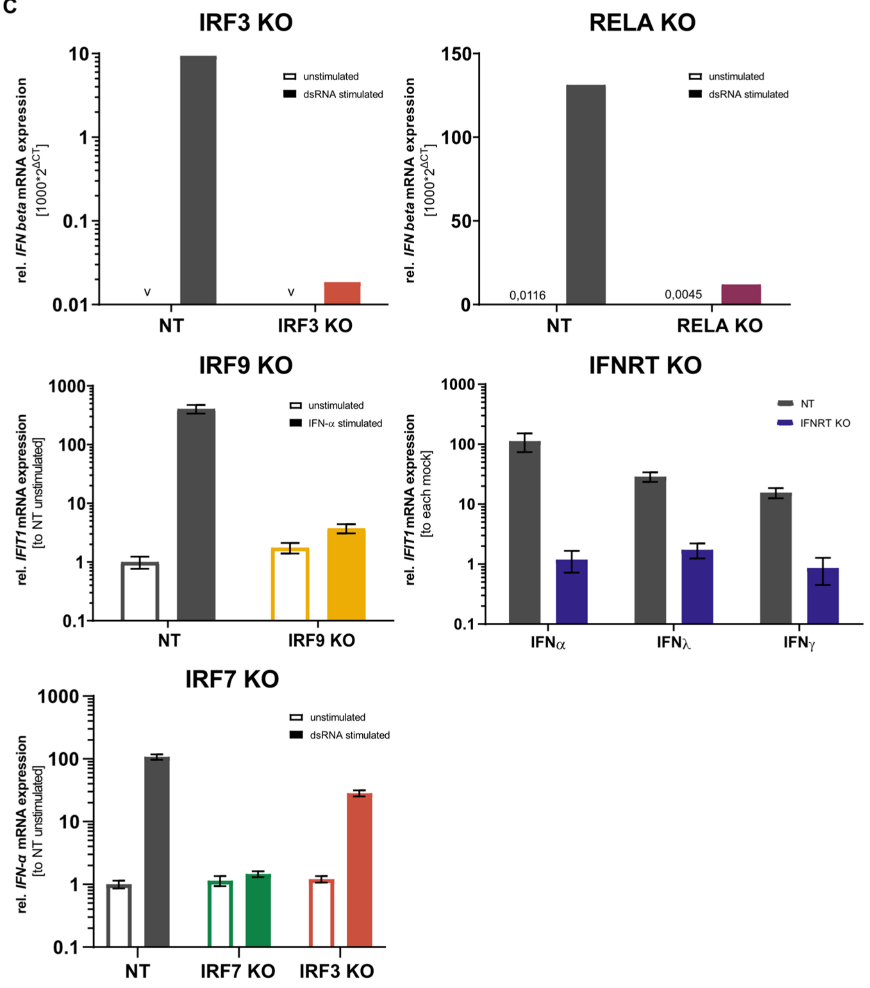

D

Name	Allele 1	Allele 2	Allele 3
IRF1	75 bp deletion	44 bp deletion	23 bp deletion
IRF2	29 bp deletion	2 bp deletion	1 bp insertion
IRF3	2 bp deletion	2 bp insertion	-
IRF5	14 bp deletion	14 + 2 bp deletion	14 + 1 bp insertion
IRF7	18 bp deletion	9 bp deletion	-
IRF9	7 bp deletion	1 bp insertion	-
RELA	1 bp insertion	-	-
RIG-I	7 bp deletion	5 bp deletion	-

Supplementary Figure S1. Validation of A549 Knockout Clones Immunoblots of A549$^{\text{IRF (as indicated) KO}}$, A549$^{\text{RELA KO}}$ and A549$^{\text{RIG-I KO}}$ with calnexin as loading control. Protein levels are shown after (A) IFN-α2a or (B) RVFVΔNSs_RLuc stimulation for 16 hours. (C) Transcript levels of IFN-α, INF-β or IFIT1 were assessed after target specific stimulation for IRF3, IRF7, IRF9, RELA and IFNAR1/IFNLR/IFNGR1 by qRT-PCR. Data show the mean ± SD of three technical replicates. (D) Table shows Indels around the sgRNA binding region detected by next generation sequencing of the indicated KO clones. A549 cells are hypotriploid / aneuploid and accordingly, for most cell clones three differently edited alleles were detected. “-“ indicates no (second or) third allele was detected, either due to identical editing of more than one locus, or due to a lack of additional alleles. In no case, wildtype allele sequences could be detected.

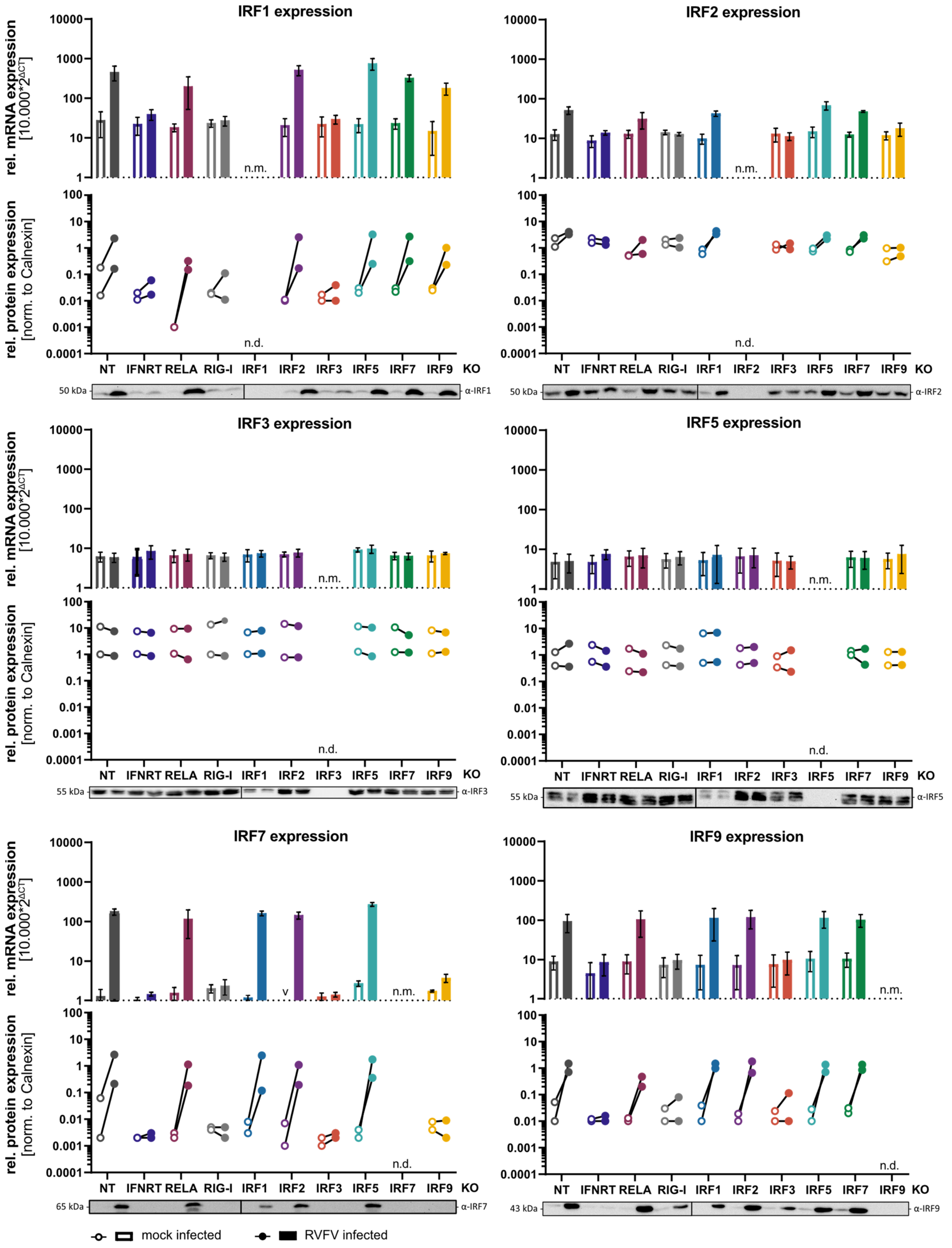

Supplementary Figure S2. Knockout validation and mutual dependency of IRFs (RVFV) A549^{NT}, A549$^{IRF\ (as\ indicated)\ KO}$, A549$^{RIG\text{-}I\ KO}$, A549$^{RELA\ KO}$ and A549$^{IFNALGR\ KO}$ cells were infected with Rift Valley Fever Virus harboring a *Renilla* luciferase in place of NSs (RVFVΔNSs-R-Luc) at an MOI of 0.01 for 16 hours. Relative IRF1, 2, 3, 5, 7 and 9 mRNA levels were measured by qRT-PCR and normalized to NT mock infected. Protein expression of the indicated samples was determined by immunoblot analysis using calnexin as loading control. Data show the mean ± SD of two (immunoblot) or three (qRT-PCR) independent experiments.

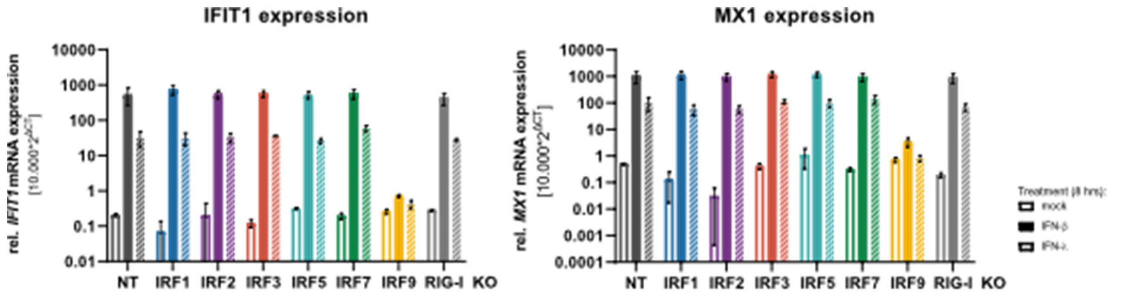

Supplementary Figure S3. Impact of IRFs on ISG induction A549^{NT}, A549$^{IRF\ (as\ indicated)\ KO}$ and A549$^{RIG\text{-}I\ KO}$ cells were stimulated with IFN-β (500 IU/ml) or IFN-λ (5 ng/ml) for 8 hours. Relative IFIT1 and MX1 mRNA levels were measured by qRT-PCR. Data show the mean ± SD of three independent experiments.

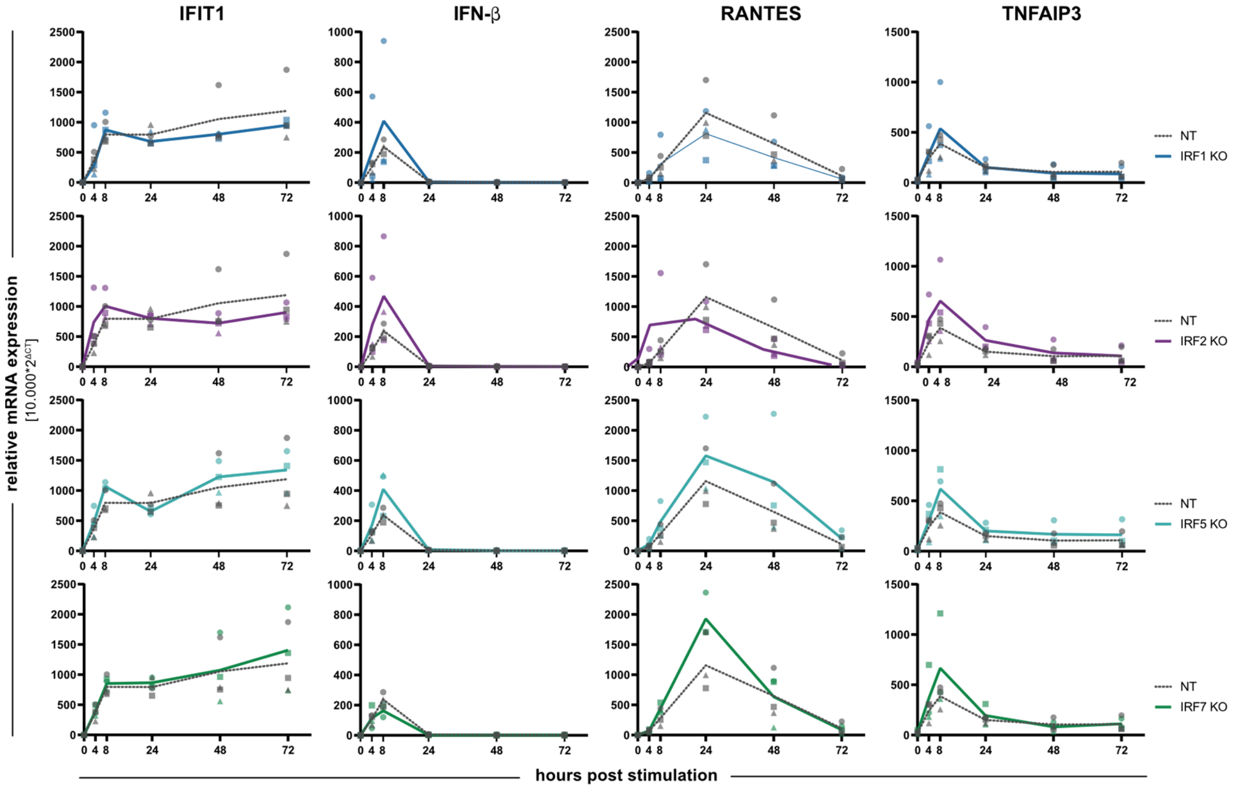

Supplementary Figure S4. Impact of IRFs on RIG-I-dependent and RIG-I-independent signaling A549^{NT} and A549$^{IRF\ (as\ indicated)\ KO}$ cells were stimulated by transfection of 5'ppp-dsRNA (2 ng/well). Relative IFIT1, IFNB, CCL5 and TNFAIP3 mRNA levels were measured at 4, 8, 24, 48 and 72 hours post stimulation by qRT-PCR. Data show individual data points and a curve representing the mean of three independent experiments.

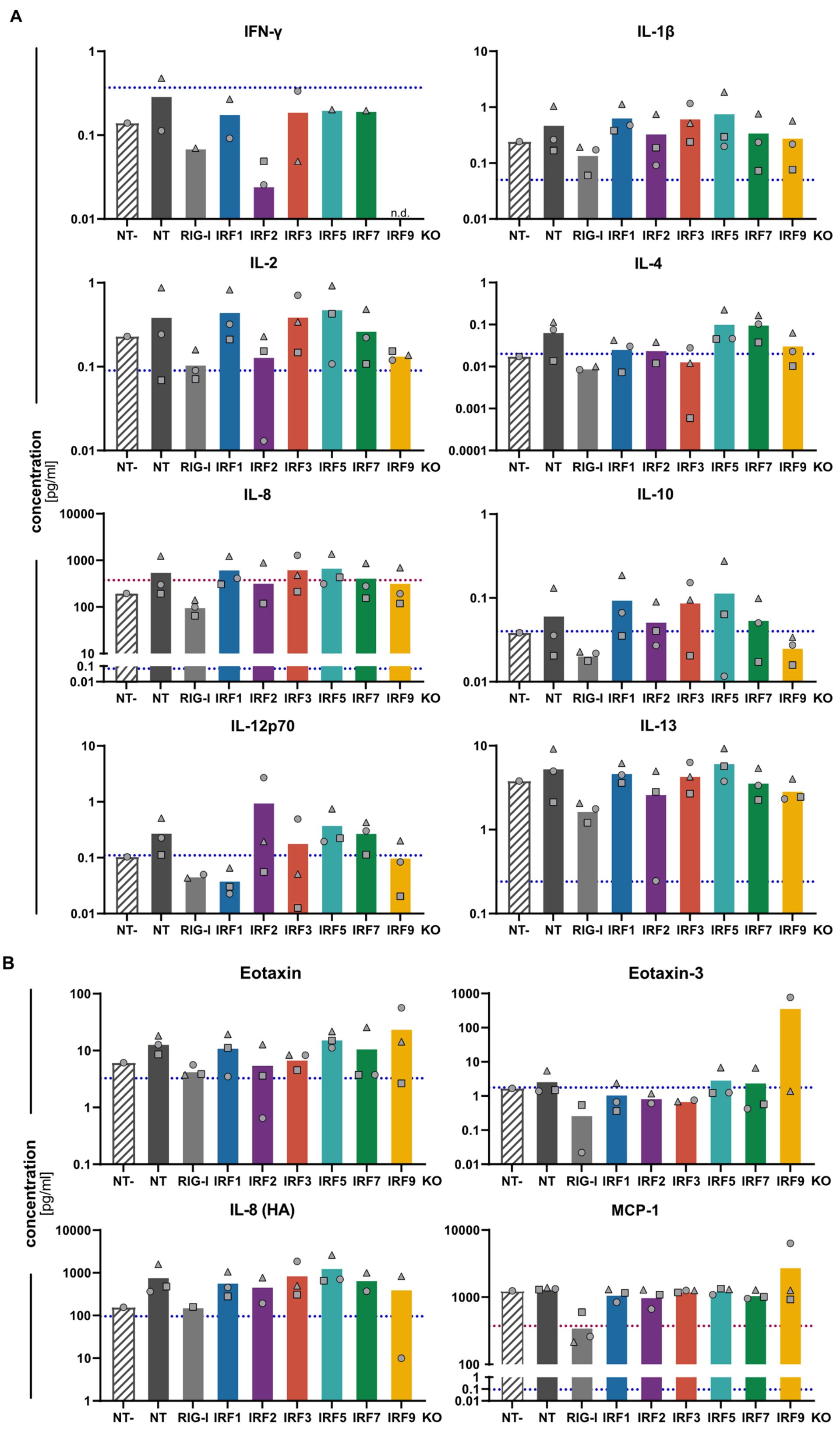

Supplementary Figure S5. Proinflammatory cytokine and chemokine profiles of IRF KO cell lines after RIG-I-specific stimulus A549^{NT}, A549$^{\text{IRF (as indicated) KO}}$ and A549$^{\text{RIG-I KO}}$ cells were stimulated by transfection of 5′ppp-dsRNA (1 ng/well) for 24 hours. Concentrations of indicated (A) proinflammatory cytokines and (B) chemokines were assessed by an electroluminescent multiplex assay of the MSD platform. NT- stands for the unstimulated control. Dashed line in blue indicates the lower limit of detection, whereas the dashed line in purple indicates the upper limit of detection of each analyte. Symbols not represented in the figure imply that the results for those measurements were zero. Data show individual data points and a bar representing the mean of three independent experiments.

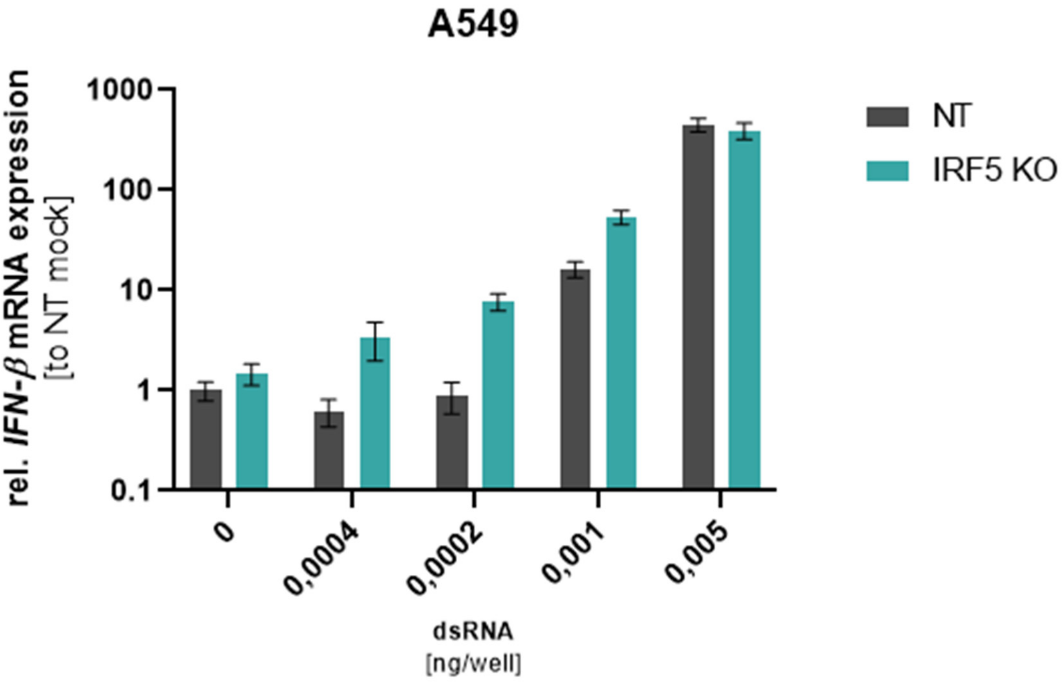

Supplementary Figure S6. IFN-β mRNA expression upon IRF5 KO To confirm slightly increased IFN-β levels upon IRF5 KO, A549[IRF5 KO] cells were transfected with a titration of RIG-I ligand 5′ppp-dsRNA in the indicated concentrations. IFN-β mRNA levels were determined 16 hours post transfection by qRT-PCR. Graph shows the mean and standard deviation of three technical replicates.

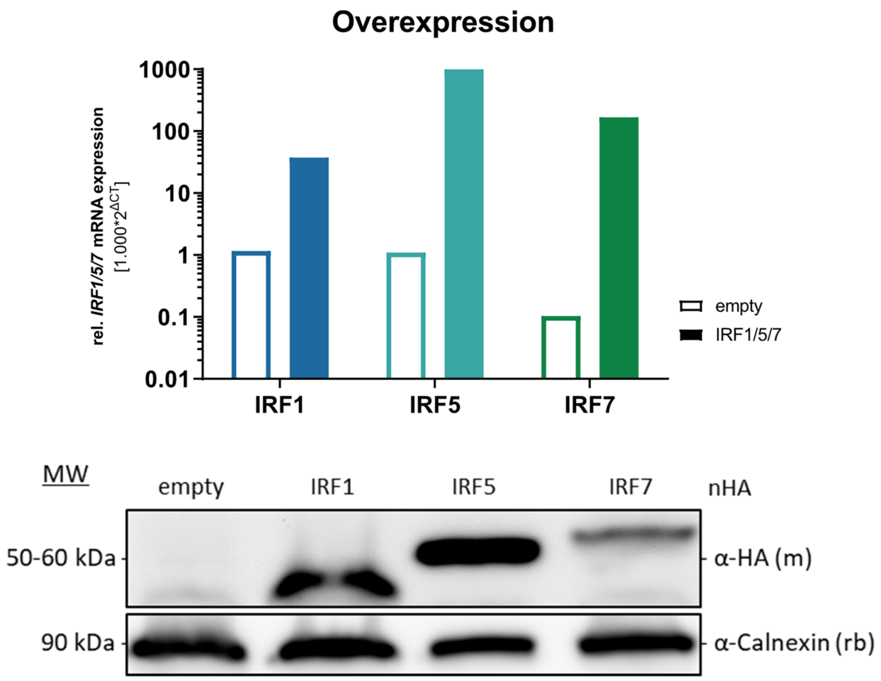

Supplementary Figure S7. Validation of A549 IRF1, IRF5, and IRF7 overexpression cell lines IRF1, IRF5, and IRF7 transcripts were measured in empty vector control and overexpression cell lines by quantitative RT-PCR. Protein levels of HA-tagged empty vector control and IRF1, IRF5, and IRF7 overexpression cell lines were determined by western blot analysis.
